# STDP in a Bistable Synapse Model Based on CaMKII and Associated Signaling Pathways

**DOI:** 10.1371/journal.pcbi.0030221

**Published:** 2007-11-30

**Authors:** Michael Graupner, Nicolas Brunel

**Affiliations:** 1 Université Paris Descartes, Laboratoire de Neurophysique et Physiologie, Paris, France; 2 CNRS, UMR 8119, Paris, France; 3 Max-Planck-Institut für Physik komplexer Systeme, Dresden, Germany; University College London, United Kingdom

## Abstract

The calcium/calmodulin-dependent protein kinase II (CaMKII) plays a key role in the induction of long-term postsynaptic modifications following calcium entry. Experiments suggest that these long-term synaptic changes are all-or-none switch-like events between discrete states. The biochemical network involving CaMKII and its regulating protein signaling cascade has been hypothesized to durably maintain the evoked synaptic state in the form of a bistable switch. However, it is still unclear whether experimental LTP/LTD protocols lead to corresponding transitions between the two states in realistic models of such a network. We present a detailed biochemical model of the CaMKII autophosphorylation and the protein signaling cascade governing the CaMKII dephosphorylation. As previously shown, two stable states of the CaMKII phosphorylation level exist at resting intracellular calcium concentration, and high calcium transients can switch the system from the weakly phosphorylated (DOWN) to the highly phosphorylated (UP) state of the CaMKII (similar to a LTP event). We show here that increased CaMKII dephosphorylation activity at intermediate Ca^2+^ concentrations can lead to switching from the UP to the DOWN state (similar to a LTD event). This can be achieved if protein phosphatase activity promoting CaMKII dephosphorylation activates at lower Ca^2+^ levels than kinase activity. Finally, it is shown that the CaMKII system can qualitatively reproduce results of plasticity outcomes in response to spike-timing dependent plasticity (STDP) and presynaptic stimulation protocols. This shows that the CaMKII protein network can account for both induction, through LTP/LTD-like transitions, and storage, due to its bistability, of synaptic changes.

## Author Summary

Learning and memory have been hypothesized to occur thanks to synaptic modifications. The efficacy of synaptic transmission has been shown to change as a function of correlated activity between presynaptic and postsynaptic neurons. Long-lasting synaptic modifications can occur in both directions (long-term potentiation (LTP) and long-term depression (LTD)). Recent experiments suggest that these synaptic changes are all-or-none switch-like changes. This would mean that only two stable states of synaptic transmission efficacy exist, i.e*.,* a low state, or “switched off”, and a high state, or “switched on”. LTP would correspond to switching on the synapse and LTD to switching off. We propose a realistic biochemical model of protein–protein interactions which exhibits two stable states. We then investigate conditions under which the model exhibits transitions between the two stable states. We show that experimental stimulation protocols known to evoke LTP and LTD lead to corresponding transitions in the model. This work supports the idea that the investigated intracellular protein network has a role in both induction and storage of synaptic changes, and hence in learning and memory storage.

## Introduction

Synaptic plasticity is thought to underlie learning and memory, but the mechanisms by which changes in synaptic efficacy are induced and maintained over time are still unclear. Numerous experiments have shown how synaptic efficacy can be increased (long-term potentiation, LTP) or decreased (long-term depression, LTD) by spike timing of presynaptic and postsynaptic neurons [1,2], presynaptic firing rate [3,4], or presynaptic firing paired with postsynaptic holding potential [5]. These experiments have led to phenomenological models that capture one or several of these aspects [6–14]. However, these models tell us nothing about the biochemical mechanisms of induction and maintenance of synaptic changes. The question of the mechanisms at the biochemical level has been addressed by another line of research work originating from early work by Lisman (1985) [15]. Models at the biochemical level describe enzymatic reactions of proteins in the postsynaptic density (PSD) [15–19]. These proteins form a network with positive feedback loops that can potentially provide a synapse with several stable states—two, in the simplest case—providing a means to maintain the evoked changes. Hence, synapses in such models are similar to binary switches, exhibiting two stable states, an UP state with high efficacy, and a DOWN state with low efficacy. The idea of binary synapses is supported by recent experiments on CA3-CA1 synapses [20–22].

One of the proposed positive feedback loops involves the calcium/calmodulin-dependent protein kinase II (CaMKII) kinase-phosphatase system [15–19]. CaMKII activation is governed by Ca^2+^/calmodulin binding and is prolonged beyond fast-decaying calcium transients by its autophosphorylation [23]. Autophosphorylation of CaMKII at the residue theronine-286 in the autoregulatory domain (Thr^286^) occurs after calcium/calmodulin binding and enables the enzyme to remain autonomously active after dissociation of calcium/calmodulin [24] (see Materials and Methods). In turn, as long as CaMKII stays activated it is reversibly translocated to a postsynaptic density (PSD)-bound state where it interacts with multiple LTP-related partners structurally organizing protein anchoring assemblies and therefore potentially delivering α-amino-3-hydroxyl-5-methyl-4-isoxazole-propionate acid (AMPA) receptors to the cell surface [23,25–28]. The direct phosphorylation of the AMPA receptor GluR1 subunit by active CaMKII enhances AMPA channel function [29,30]. The network involving CaMKII is particularly appealing in terms of learning and memory maintenance since N-methyl-D-aspartate receptor (NMDA-R)-dependent LTP requires calcium/calmodulin activation of CaMKII, potentially expressed by the phosphorylation level or the number of AMPA receptors, or both [19,27,28,31–33]. However, the role of CaMKII beyond LTP induction remains controversial [34–36]. Finally, there is experimental evidence for the involvement of proteins associated with CaMKII activity (cyclic adenosine monophosphate (cAMP)–regulated protein kinase A (PKA), protein phosphatase 1 (PP1), and calcineurin) in LTP and LTD [37–40]. We emphasize that multiple mechanisms supporting LTP/LTD induction and expression are likely to be present in synapses of different regions—we focus here on synapses for which the above statements have been shown to apply, e.g., the CA3-CA1 Schaffer collateral synapse (see review by Cooke and Bliss [41]).

Modeling studies have shown that a system including CaMKII and associated pathways could be bistable in a range of calcium concentrations including the resting level—a necessary requirement for the maintenance of long-term changes [15,17,18,42]. In such models, the two states correspond to two stable phosphorylation levels of the CaMKII protein for a given calcium concentration, i.e., a weakly (DOWN) and a highly phosphorylated state (UP). A transition from the DOWN to the UP state which could underlie long-term potentiation (LTP) can be induced by a sufficiently large and prolonged increase in calcium concentration. However, the opposite transition which could underlie depotentiation or LTD only occurs under unrealistic conditions, for example decrease of calcium concentration below resting level. Furthermore, it has not been considered how these biochemical network models behave in response to calcium transients evoked by experimental protocols that are known to induce synaptic plasticity such as STDP, which has been shown to rely on kinase (CaMKII) and phosphatase (calcineurin) activation [43]. Rubin et al. reproduce experimental results on STDP using a model detector system which qualitatively resembles the protein network influencing CaMKII, but this model does not exhibit bistability [44]. Other studies on biochemical signal transduction pathways including CaMKII showed that the AMPA receptor activity can reproduce bidirectional synaptic plasticity as a function of calcium [45,46]. However, realistic stimulation protocols were not investigated in these models, and again they do not show bistability.

In this paper, we consider a realistic model of protein interactions associated with CaMKII autophosphorylation through calcium/calmodulin and dephosphorylation by protein phosphatase 1 in the PSD. We first study the steady-state phosphorylation properties of CaMKII with respect to calcium and changing levels of PP1 activity. Conditions are elaborated for which the system allows for “LTP” and “LTD” transitions in reasonable ranges of calcium concentrations. We then demonstrate the ability of the CaMKII system to perform LTP- or LTD-like transitions in response to STDP stimulation protocols. We expose the CaMKII system to calcium transients evoked by pairs of presynaptic and postsynaptic spikes with a given time lag and show that short positive time lags evoke transitions from the DOWN to the UP state and short negative time lags lead to transitions from the UP to the DOWN state. We demonstrate furthermore that the CaMKII model qualitatively reproduces experimental plasticity outcomes for presynaptic stimulation protocols. Finally, we consider the transition behavior in response to purely presynaptic or postsynaptic spike-pair stimulation protocols.

## Results

**Figure 1:**
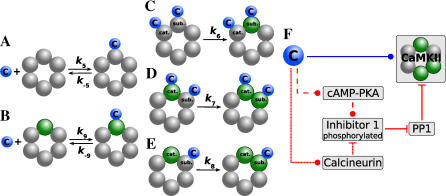
Schematic Representation of Calmodulin Binding to a CaMKII Subunit, Intersubunit Phosphorylation Steps, and the Protein Signaling Cascade (A–E) Show calcium/calmodulin binding and phosphorylation reactions of a ring of six functionally coupled subunits of the CaMKII holoenzyme. A gray subunit stands for dephosphorylated and a green subunit surrounded by a dotted line for phosphorylated. (A,B) The calcium/calmodulin complex—C, blue circle—can bind to a dephosphorylated (A) or a phosphorylated subunit (B) with dissociation constants *K*_5_ = *k*_−5_ / *k*_5_ or *K*_9_ = *k*_−9_ / *k*_9_, respectively. Note that the calmodulin binding (A,B) and the autophosphorylation steps (shown in C–E) are assumed to take place independently of the phosphorylation state of other subunits in the ring (here depicted as dephosphorylated, i.e*.*, in gray). Subunits shown in dotted gray can be either dephosphorylated or phosphorylated. (C–E) The three possible intersubunit phosphorylation steps: in all three cases, the catalytic subunit and the substrate subunit are labeled with cat. and sub., respectively. Unlabeled subunits are depicted as dephosphorylated, but the three phosphorylation steps are assumed to proceed independently of their phosphorylation state. (C) Initiation step: calmodulin has to bind to the two interacting subunits, i.e., to the substrate and the catalyst, in order to phosphorylate the substrate subunit at Thr^286^ (shown in green and surrounded by a dotted line). (D) Calmodulin is bound to the phosphorylated catalyst and the subunit to be phosphorylated. (E) The phosphorylated subunit stays active as catalyst after calmodulin dissociation and phosphorylates the substrate subunit bound with calmodulin. *k*_6_, *k*_7_, and *k*_8_ denote the respective autophosphorylation rates of the three steps described above. (F) Protein signaling cascade governing PP1 activity. Interactions shown with a circle at the end of a line indicate stimulation—whereas lines ending with a bar stand for inhibition of target activity. Calcium/calmodulin—C, blue circle—directly phosphorylates CaMKII (blue line). Furthermore, the dephosphorylation of CaMKII by protein phosphatase 1 (PP1) is indirectly controlled by calcium/calmodulin via a protein signaling cascade (red lines). Calcium/calmodulin-directed phosphorylation of inhibitor 1 via cyclic-AMP and PKA increases CaMKII activity by inhibiting PP1. This CaMKII stimulating pathway is depicted in red dashed lines. On the contrary, activation of calcineurin activates PP1 by dephosphorylating inhibitor 1, which in turn leads to increased CaMKII dephosphorylation. This pathway, shown in red dotted lines, decreases CaMKII activity.

We investigate in this paper a realistic model for the protein network of the postsynaptic density, focusing on the pathways affecting the phosphorylation dynamics of CaMKII localized in the PSD. The model describes the calcium/calmodulin-dependent autophosphorylation of CaMKII. Phosphorylation of a CaMKII subunit by its neighboring subunit requires calcium/calmodulin to bind to the substrate subunit. The catalytic subunit is active if bound to Ca^2+^/calmodulin, or phosphorylated (see Figure 1A–1E). Dephosphorylation of phosphorylated CaMKII subunits by PP1 in the PSD is implemented according to the Michaelis-Menten scheme. We also take into account how calcium/calmodulin influences PP1 activity via a protein signaling cascade. PP1 is inhibited by phosphorylated inhibitor 1 (I1). The phosphorylation level of inhibitor 1 is in turn controlled by the balance between a pathway phosphorylating I1 (through cAMP–PKA) and a pathway dephosphorylating I1 (through calcineurin). Therefore, calcineurin activation by calcium/calmodulin increases PP1 activity, while calcium/calmodulin-dependent activation of the cAMP–PKA pathway decreases PP1 activity (Figure 1F). Finally, we model postsynaptic calcium and postsynaptic membrane potential dynamics induced by presynaptic and postsynaptic spikes in order to investigate the effects of spike-induced calcium transients on the dynamics of the system. Details of the model can be found in the Materials and Methods section.

### Bistability of the CaMKII system with Constant PP1 Activity

In this and the following section we investigate how the *steady-state* values of the total concentration of phosphorylated CaMKII subunits, *S*_active_, depend on the concentration of calcium and the dephosphorylation activity. We also study how the steady-state behavior changes with the number of interacting subunits in the cluster. We start by exploring Ca^2+^/calmodulin-stimulated autophosphorylation of CaMKII at a fixed dephosphorylation activity. This will allow us later to better understand how the parameters of the signaling cascade controlling dephosphorylation activity affect the phosphorylation behavior of CaMKII. To do this, we set the PP1 dephosphorylation activity to a constant, independent of the calcium concentration (this is equivalent to removing the red lines in Figure 1F except for the interaction between CaMKII and PP1). The PP1 dephosphorylation activity is the product of *k*_12_, the maximal dephosphorylation rate, and *D*, the free PP1 concentration (see Equation 6).

**Figure 2:**
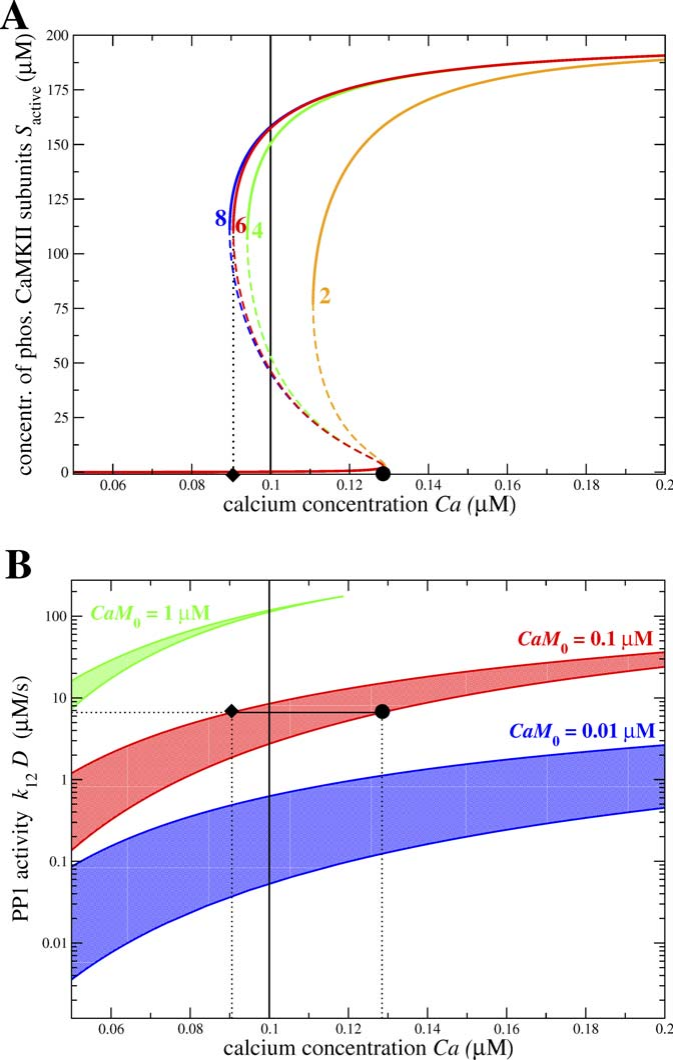
CaMKII Bistability Properties with Constant PP1 Activity (A) Bistability of the steady-state concentration of phosphorylated CaMKII subunits as a function of calcium concentration, for various numbers of subunits: the steady-state concentration of phosphorylated CaMKII subunits, *S*_active_, is shown for different numbers of subunits in an interacting cluster (subunit number indicated). Dashed thin lines characterize unstable steady-states and full lines show stable steady-states. The left-hand calcium boundary of the bistable region for the six-subunit model is indicated by the diamond and the right-hand boundary by the circle. In all cases, the total concentration of subunits is 200 μM, i.e., *CaMKII*_0_ = 50 μM for the two-subunit case, *CaMKII*_0_ = 25 μM for the four-subunit case, *CaMKII*_0_ = 16.67 μM for the six-subunit, and *CaMKII*_0_ = 12.5 μM for the eight-subunit case. The total level of calmodulin, *CaM*_0_, is 0.1 μM for all cases. The vertical full line shows the position of the calcium resting concentration in (A) and (B). The PP1 activity is kept constant at (*k*_12_ · *D*) = 6.648 μM/s. (B) Boundaries of the bistable region in the PP1 activity—calcium concentration plane for different levels of calmodulin with the six-subunit model: lines of any given color depict the location of the left-hand (upper line) and the right-hand (lower line) boundaries of the bistable region with respect to the PP1 activity. Shaded areas between both boundaries mark the regions of bistability in the PP1 activity—calcium plane. Different colors correspond to different levels of calmodulin as indicated in the panel. The diamond illustrates the position with respect to calcium of the left-hand boundary (*Ca* = 0.091 μM) and the circle of the right-hand boundary (*Ca* = 0.129 μM) of the bistable region for parameters as for the six-subunit case in (A) (*CaM*_0_ = 0.1 μM, (*k*_12_·*D*) = 6.648 μM/s). See Table 1 for other parameters.

Figure 2A shows the steady-state concentration of phosphorylated CaMKII subunits as a function of the calcium concentration for 2, 4, 6, and 8 functionally connected subunits in the CaMKII cluster. The graphs show that in all cases there exists a range of calcium concentration for which the system is bistable (region between the diamond and the circle in the case of the six-subunit model). In the bistable region, three steady-states are present. The top and the bottom steady-states (depicted by the thick full lines) are stable, whereas the intermediate one (dashed thin lines) is unstable. The branch of unstable steady-states separates the basins of attraction of the highly and the weakly phosphorylated stable steady-states. This means that the system will converge to the UP state if it is initially above this line, while it will converge to the DOWN state if it is below this line. As in other studies on CaMKII bistability, the bistable phosphorylation behavior emerges from the combination of strong cooperativity of CaMKII autophosphorylation and the saturation of the per-subunit dephosphorylation rate, *k*_10_ (see Equation 6 in Materials and Methods ), at high phosphorylation levels [15,17,18]. This saturation arises from the Michaelis-Menten approach employed to describe dephosphorylation, which is valid if the enzyme (PP1) is present in small amounts compared to the substrate (phosphorylated subunits). This is plausible since the CaMKII protein is localized at high concentrations in the PSD [24,27,47].

**Table 1:**
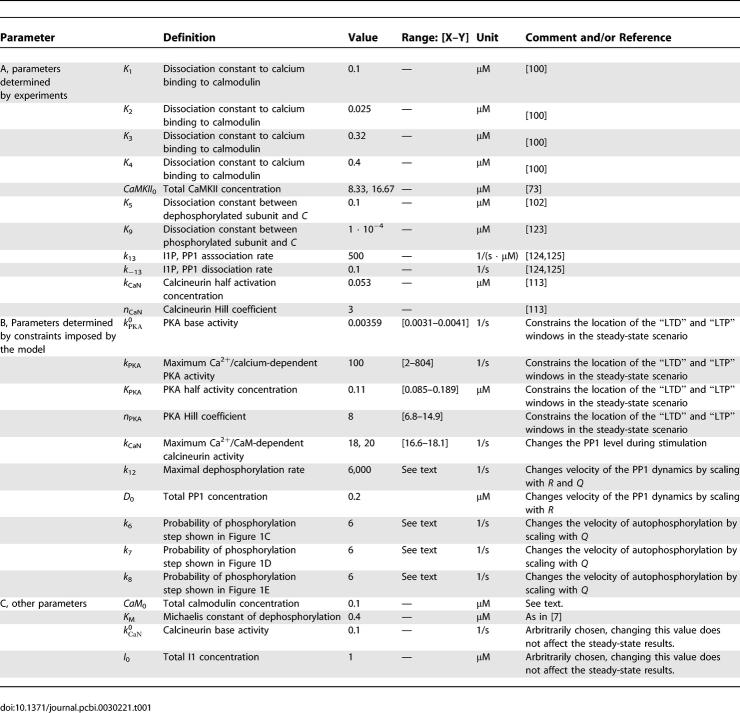
Parameters of the Model

Figure 2A demonstrates that the increasing saturation of the per-subunit dephosphorylation rate with increasing number of interacting subunits in the holoenzyme ring plays a crucial role in the extent of the bistable region. Whereas the difference between the two- and the four-subunit model is very pronounced, increasing further the number of subunits has less and less impact on the size of the bistable region—it still increases substantially when this number goes from four to six, but there is almost no noticeable difference between the six- and the eight-subunit model. The effect of the number of subunits on the extent of the bistable region is mainly due to an increase in the range of stability of the UP state with increasing subunit number, since the stronger saturation of the per-subunit dephosphorylation rate becomes apparent in the highly phosphorylated state only (see Equation 6). On the other hand, the range of stability of the DOWN state is essentially unaffected by the number of subunits, since it is mostly controlled by the balance between the dephosphorylation rate and the probability of the initiation step to occur. Interestingly, experimental data indicate that the number of functionally coupled subunits in a CaMKII holoenzyme ring is six [48–50], which could be a good compromise between having both a relatively small number of subunits and a large bistability range. In the following, we consider exclusively a model with six subunits.

How the location and the extent of the bistable region changes with respect to the PP1 dephosphorylation activity is shown in Figure 2B. The curves depict the boundaries of the bistable region for the six-subunit model in the PP1 activity—calcium concentration plane, for three values of the total calmodulin concentration, *CaM*_0_ (indicated by the three different colors). For each value of *CaM*_0_, the colored area shows the bistable region in which the UP and the DOWN states coexist. Above the colored area, only the DOWN state is present, while below that area only the UP state is present. The resting calcium concentration can be included in the bistable region, provided the PP1 activity is chosen accordingly (e.g., (*k*_12_·*D*) = 6.648 μM/s for *CaM*_0_ = 0.1 μM in Figure 2B).

### “LTD Window” in a Model with Ca-Dependent PP1 Activity via Protein Signaling Cascade Including PKA and Calcineurin

The right-hand boundary of the bistable region in Figure 2A corresponds to a down-to-up switching threshold: if the calcium concentration increases persistently above this level, the CaMKII will converge from a weakly phosphorylated to a highly phosphorylated state (down-to-up switching). Hence, we define the range above this right-hand bifurcation point “LTP window”. It corresponds to high calcium concentrations, consistent with experimental data on the range of calcium concentrations leading to LTP.

The available experimental data also suggest that (i) at resting calcium concentrations, no transitions should occur (both UP and DOWN states should be stable), (ii) for intermediate calcium concentrations (higher than resting concentration, but lower than the down-to-up switching threshold), LTD transitions should occur. This would happen if the UP state was no longer stable in an intermediate range of calcium concentrations—in such a scenario, the UP state would be stable in two disconnected regions, one around resting calcium concentration, and the other one at high calcium concentrations. The region where the UP state would not be stable could be called “LTD window” since the system would exhibit LTD (up-to-down switching) whenever the calcium concentration stays in that region for a sufficiently long time, i.e., the CaMKII would converge from a highly phosphorylated state to a weakly phosphorylated state in this range of calcium. The scenario depicted in Figure 2 seems at odds, however, with this picture.

How can the steady-state picture of Figure 2A be modified to obtain such an LTD window? A possible scenario is to take into account the protein signaling cascade governing PP1 dephosphorylation activity in a calcium/calmodulin-dependent manner (see Figure 1F). In this way, the active concentration of PP1, *D*, changes with calcium, and the region of bistability is no longer defined by a horizontal line in Figure 2B. Rather, the location and extent of the LTD and the LTP windows are given by the intersections of the curve describing how the steady-state PP1 activity changes with calcium concentration with the curves specifying the location of the left- and the right-hand boundary of the bistable region in the PP1 activity–calcium concentration plane.

**Figure 3:**
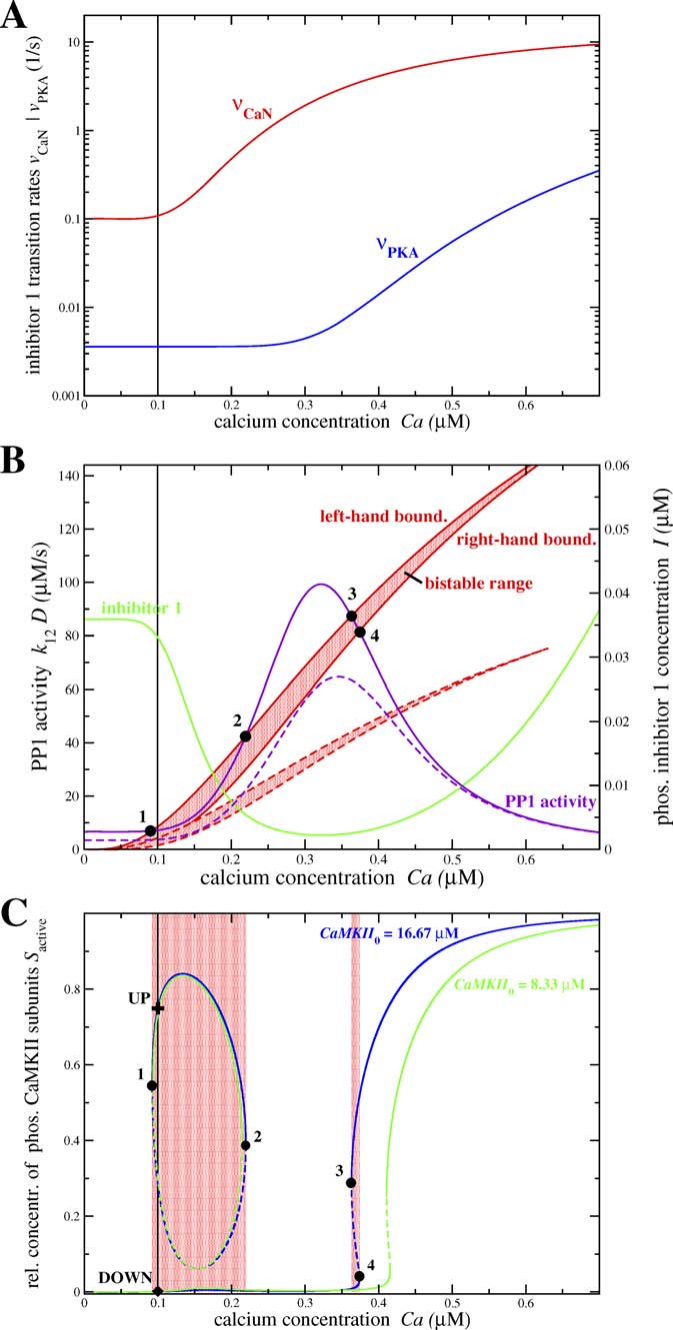
Ca^2+^-Dependent PP1 Activity via Protein Signaling Cascade and Phosphorylated CaMKII Subunit Concentration Steady-States (A) Phosphorylation and dephosphorylation rate of inhibitor 1 as functions of the calcium concentration: the red line shows the calcineurin activity dephosphorylating inhibitor 1, *v*_CaN_(*C*), as a function of calcium, and the blue line shows the PKA activity leading to phosphorylation of I1, *v*_PKA_(*C*). Both rates are given by Equation 9, in which the calcium/calmodulin complex concentration, *C*, is in equilibrium with the present calcium concentration. The total calmodulin concentration is Ca*M*_0_ = 0.1 μM. The vertical thin full line depicts the position of the calcium resting concentration in all panels. (B) Bistable region, PP1 activity, and I1P steady-state concentration in the PP1–Ca^2+^ plane: the full lines correspond to a total CaMKII concentration of *CaMKII*_0_ = 16.67 μM, whereas dashed lines correspond to *CaMKII*_0_ = 8.33 μM. The full red lines mark the positions of the left-hand (line above) and right-hand (line below) boundaries of the bistable region (red shaded region between both lines) with respect to the PP1 activity for *CaM*_0_ = 0.1 μM. The purple and the green full lines show the calcium-dependent steady-state of the PP1 activity, (*k*_12_ · *D*), and I1P concentration, *I*, respectively, given by the rates shown in (A). The numbered points 1, 2, 3, and 4 at the intersections of the PP1 steady-state with the full red lines give the locations of the boundaries (saddle-node bifurcation points) of the bistable regions. The dashed red and purple lines depict the boundaries of the bistable region and the steady-state PP1 activity, respectively, for the case *CaMKII*_0_ = 8.33 μM (see text). (C) Steady-states of the phosphorylated CaMKII subunit concentration (*S*_active_) as a function of calcium: full lines characterize stable steady-states whereas dashed lines mark unstable steady-states. Steady-states for *CaMKII*_0_ = 16.67 μM are shown in blue and for *CaMKII*_0_ = 8.33 μM in green. Red shaded areas depict regions of bistability for the *CaMKII*_0_ = 16.67 μM case, i.e., three steady-states (two stable and one unstable steady-state) exist for a given calcium concentration. Bifurcation points are numbered as in (B). The cross (diamond) marks the position of the UP (DOWN) state at resting calcium concentration for the *CaMKII*_0_ = 16.67 μM case (see Table 1 and text for parameters, *k*_CaN_ = 18 1/s).

Figure 3B shows an example in which the steady-state PP1 activity (*k*_12_ · *D*) versus calcium concentration curve (purple line) intersects the bifurcation lines (red lines) four times, such that an LTD window emerges in a range of intermediate calcium concentrations. As Figure 3B shows, this can be obtained whenever the PP1 activity has a sufficiently large peak at some intermediate calcium concentrations. This peak has to be such that in a range of calcium concentrations, PP1 activity is above both bifurcation lines (region between intersection points **2** and **3** in Figure 3B). As discussed above, only the DOWN state is stable in this region. This PP1 peak is in turn obtained due to PKA activating at higher calcium concentration than calcineurin, since the balance between calcineurin and PKA activity determines the level of PP1 inhibition via inhibitor 1 (see Figures 1F and 3A). Hence, the peak in steady-state PP1 concentration at intermediate calcium concentrations is due to a relative increase in calcineurin activity with respect to PKA activity in this range (compare Figure 3A and 3B). To include the calcium resting concentration, *Ca*_0_ (marked by the vertical thin line in Figure 3), in a region of bistability, PP1 activity at *Ca*_0_ has to reside in between the bifurcation lines. The fourth intersection point defines the down-to-up switching threshold, i.e., the left-hand boundary of the LTP window (see point **4** in Figure 3B and 3C). The range of bistability between points marked **3** and **4** in Figure 3C emerges from the declining PP1 activity (purple line in Figure 3B) crossing the ascending range of bistability (red shaded area in Figure 3B). These opposing trends lead to a narrow range of bistability at high calcium concentrations in the example presented here since the intersections of both define the borders of the bistable region.

In practice, the location of these four intersection points can be chosen by adjusting parameters describing the calcium/calmodulin-dependent activation of PKA and calcineurin activity (see Materials and Methods section for more details). We can obtain four such parameters (PKA base and maximal activity 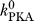 and *k*_PKA_, respectively, the PKA half activity concentration *K*_PKA_ and the PKA Hill coefficient *n*_PKA_ (see Equation 9)) by simultaneously solving four equations, i.e.*,* one for each of the four intersection points **1**,** 2**,** 3,** and **4** at *Ca* = 0.09, 0.22, 0.36, and 0.37 μM (see Figure 3B). Figure 3A displays the resulting instantaneous calcium/calmodulin-dependent phosphorylation *v*_PKA_ and dephosphorylation *v*_CaN_ rates of inhibitor 1 (see Equation 23 in Materials and Methods) leading to the steady-state PP1 concentration scenario shown in Figure 3B by the full purple line. The parameters obtained through this procedure can be found in Table 1B). Table 1B also shows the ranges of values of each parameter for which the above-described behavior is qualitatively observed. These ranges are obtained varying each parameter while keeping the remaining three constant. This shows that the system is relatively robust to parameter changes. It reacts most sensitively to changes in 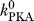 , whose value can be varied by about 14% in both directions, while the other parameters can be varied over a range of about 100% and even **∼**800% for *k*_PKA_. Note that the choice of the maximal calcineurin activity, which basically controls the height of the PP1 peak shown in Figure 3B, depends also on constraints discussed in the following section.

The system is also robust to changes in the total CaMKII concentration, as shown in Figure 3C where we compare the bifurcation diagrams for *CaMKII*_0_ = 16.67 μM (blue line) and 8.33 μM (green line) provided the PP1 activity is rescaled accordingly by using 
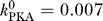
 1/s in the latter case (other parameters remain unchanged). Note that both values of *CaMKII*_0_ cover a range of CaMKII concentration that encompasses experimental estimates (∼10 μM) for the PSD [24,27,47]. The dashed lines in Figure 3B show the position of the bistable region (dashed red lines) and the steady-state PP1 activity (dashed purple line) in the PP1 activity-calcium plane for *CaMKII*_0_ = 8.33 μM and 
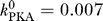
 1/s (see Table 1A and 1B for other parameters).

To summarize the results so far, the model behavior is such that: (i) the calcium resting concentration is included in a region of bistability, giving rise to two stable steady-states—DOWN and UP—at resting conditions (marked by the diamond and the cross in Figure 3C, respectively); (ii) in a region of intermediate calcium concentrations, only a weakly phosphorylated steady-state exists (the LTD window, between filled circles marked **2** and **3**); (iii) conversely, at high calcium concentrations, only the highly phosphorylated steady-state is stable (the LTP window, beyond filled circle marked **4**). This scenario is now qualitatively consistent with experimental data. Note that, in contrast with Zhabotinsky (2000), our model does not require an unrealistic high phosphorylated inhibitor 1 concentration at resting calcium concentration to have a stable highly phosphorylated CaMKII state (compare green line in Figure 3B and [17] at this concentration).

### Dynamic Response of the Model to the STDP Protocol

Up to this point, we have investigated the steady-states of the CaMKII kinase-phosphatase system as a function of the intracellular calcium concentration. In experimental conditions, however, synaptic modifications are evoked by calcium transients resulting from experimental stimulation protocols inducing synaptic plasticity. Hence, the occurrence of transitions between weakly and highly phosphorylated states in the model needs to be examined in response to such calcium dynamics. Here, we explore in which conditions the spike-timing dependent plasticity (STDP) protocol as well as presynaptic or postsynaptic stimulation protocols alone induce such transitions.

For the STDP protocol, we use a standard repetitive stimulation protocol (60 pairs at 1 Hz, see experiments by Bi and Poo [2]). Each stimulation pair consists of a presynaptic spike at time *t*_pre_ and a back-propagating postsynaptic action potential occurring at time *t*_post_ = *t*_pre_ + Δ*t*. In experimental conditions, LTD is evoked for short negative Δ *t*s, while LTP is evoked for short positive Δ *t*s [1,2,51–53].

### STDP Protocol Stimulation with Deterministic Calcium Dynamics

**Figure 4:**
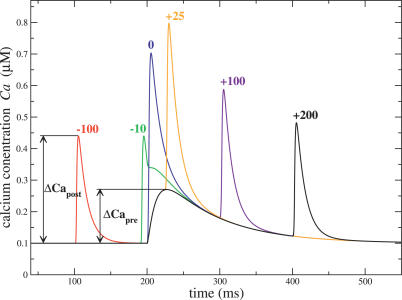
Calcium Dynamics Evoked by a Pair of a Presynaptic and a Postsynaptic Spike Occurring at Different Time Lags, Δ*t* Temporal evolution of the intracellular calcium concentration generated by the model in response to a presynaptic spike at *t*_pre_ = 200 ms and an additional postsynaptic spike at *t*_post_ = *t*_pre_ + Δ*t*, where Δ*t* is indicated at the corresponding curve in ms. The calcium amplitudes of the isolated presynaptic (Δ*Ca*_pre_ = 0.17 μM) and postsynaptic (Δ*Ca*_pre_ = 0.34 μM) responses are indicated in the panel. See Table 2 for parameters.

Figure 4 shows the time course of calcium concentration transients evoked by one pair of a presynaptic spike and a back-propagating action potential (BPAP) at different Δ *t*s. An isolated postsynaptic spike generates a calcium transient of amplitude Δ*Ca*_post_, due to opening of calcium channels induced by the depolarization caused by the BPAP. Likewise, an isolated presynaptic spike generates another calcium transient of amplitude Δ*Ca*_pre_, due to NMDA channel opening. Below, we will vary systematically the size of Δ*Ca*_pre_, keeping the ratio constant, Δ*Ca*_post_ / Δ*Ca*_pre_ = 2 [54]. See Materials and Methods for details of the model.

**Table 2:**
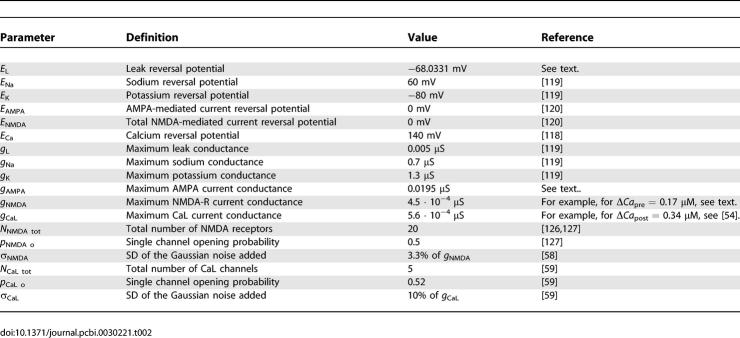
Further Parameters Used in the Model

What happens when presynaptic and postsynaptic spikes are sufficiently close together so that their respective calcium transients overlap?

#### For short negative time differences, Δ *t* < 0 ms (post before pre).

The decaying phase of the fast BPAP-evoked calcium signal overlaps in time with the long-lasting calcium transient mediated by NMDA receptors (see Figure 4 for the Δ *t* = −10 ms case). Though this temporal overlap has only a weak effect on the integral of the calcium transient induced by the pair of spikes compared to a case in which both transients do not interact at all (large positive and large negative Δ *t*), the time spent by the system in different intervals of calcium concentration does change significantly with Δ *t*. This feature largely contributes to the fact that LTD can potentially be observed at short negative Δ *t*s only (see below).

#### For positive time differences, Δ *t* > 0 ms (pre before post).

The strong depolarization by the BPAP increases drastically the voltage-dependent NMDA-R mediated calcium current, leading to a supralinear superposition of the two contributions. The ratio between the calcium peak amplitude at Δ *t* = 10 ms and the linear sum of individual presynaptically and postsynaptically evoked calcium transients is about 1.6, consistent with experimental data [55]. This supralinearity explains to a large extent the occurrence of LTP at short positive Δ *t* and also prevents LTD transitions at large positive Δ *t* protocols (see discussion below). The repetitive presentation (60 times at 1 Hz) of the presynaptic and postsynaptic spike pair produces repetitively the calcium transient shown in Figure 4 for a few examples of Δ *t*.

### LTP and LTD-Like Transitions as a Function of Δ *t*

**Figure 5:**
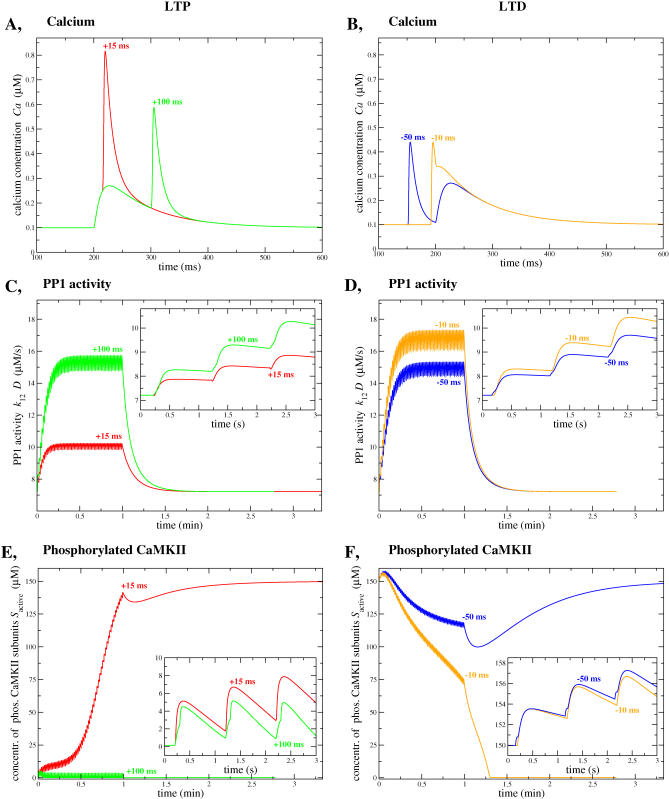
Time Course of Calcium, PP1 Activity, and Phosphorylated CaMKII Subunit Concentration During STDP Stimulation Protocols with Δ *t* = −50, −10, 15, and 100 ms (A,C,E) Show the dynamics during STDP stimulation protocols with Δ *t* = 15 ms (red lines) and Δ *t* = 100 ms (green lines), with the system initially in the DOWN state. (B,D,F) The CaMKII system is initially in the UP state and is subjected to stimulation protocols with Δ *t* = −50 ms (blue lines) and Δ *t* = −10 ms (orange lines). Note that the time course of calcium dynamics (A,B) is shown for one spike-pair presentation only. The PP1 and *S*_active_ time course (C–F) is shown for the full-stimulation protocol with 60 spike-pair presentations at 1 Hz and until the system converges to the final steady-state. The insets in panel (C–F) display the dynamics on a shorter time scale (first three spike pairs in the stimulation protocol). In this figure: *k*_CaN_ = 18 1/s, Δ*Ca*_pre_ = 0.17 μM. The PP1 steady-state activity is 7.21 μM/s at *Ca*_0_ = 0.1 μM (compare Figure 3B).

**Figure 6:**
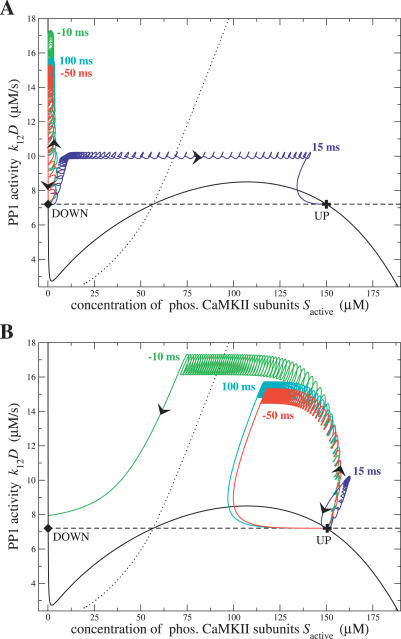
Dynamics of the System in the PP1 Activity–Phosphorylated CaMKII Subunit Concentration Phase-Plane (A,B) Show the trajectories in the (*k*_12_ · *D*) − *S*_active_ phase-plane during STDP stimulation protocols with Δ *t* = −50 ms (red), Δ *t* = −10 ms (green), Δ *t* = 15 ms (blue), and Δ *t* = 100 ms (cyan), and until the system reaches the final steady-state. (A) System initially in the DOWN state (diamond). (B) System initially in the UP state (cross). The black arrows indicate the direction of motion of the system along the trajectory for some examples. The full and the dashed black line depict the *S*_active_ and the (*k*_12_ · *D*) nullclines at resting conditions (*Ca*_0_ = 0.1 μM), respectively. Therefore, the intersections of both nullclines mark the positions of the steady-states of the system: two stable—the DOWN (diamond) and the UP (cross) state—and one unstable at *S*_active_ ≈ 56.8 μM. Note that the PP1 activity nullcline (dashed black line) is independent of *S*_active_ (see Equations 24 and 25), whereas the *S*_active_ nullcline (full black line) is dependent on (*k*_12_ · *D*) and *S*_active_ (see Equations 7–20). The separatrix, separating the basins of attraction of the two stable steady-states is shown as a dotted black line (see Table 1 for parameters, *k*_CaN_ = 18 1/s, Δ*Ca*_pre_ = 0.17 μM).

**Figure 7:**
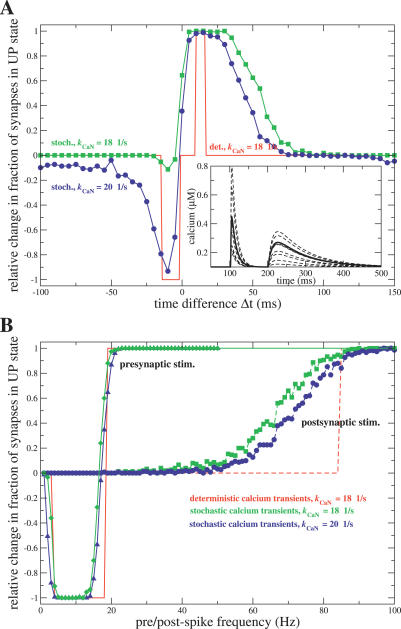
Synaptic Modifications in Response to STDP, Purely Presynaptic, or Purely Postsynaptic Stimulation Protocols (A) The relative change in the fraction of synapses in the UP state in response to deterministic (red line) and stochastic (green and blue lines with symbols) STDP stimulation protocols is shown as a function of Δ *t*. 0 means no net change in number of synapses in the UP state, positive relative change means a net increase of synapses in the UP state, and a negative relative change means a net increase of synapses in the DOWN state. Stochastic stimulation results are shown for *k*_CaN_ = 18 1/s (green line with squares, same value as in deterministic case) and *k*_CaN_ = 20 1/s (blue line with circles). The inset shows some example calcium transients (dashed lines) of the stochastic stimulation protocol evoked by a spike pair with Δ *t* = −100 ms and *t*_pre_ = 200 ms. The avergage calcium transient is shown by the full line and is the same as the red curve in Figure 4. (Δ*Ca*_pre_ = 0.17 μM.) (B) Relative change in the fraction of synapses in the UP state after purely presynaptic, or purely postsynaptic stimulation protocols, as a function of frequency: the stimulation protocol consists of 60 presynaptic (full lines) or postsynaptic spikes (dashed lines) at a given frequency. Red lines: deterministic stimulation, *k*_CaN_ = 18 1/s. Green lines with diamonds or squares: stochastic presynaptic or postsynaptic stimulations, respectively, with *k*_CaN_ = 18 1/s. Blue line with triangles or circles: stochastic presynaptic or postsynaptic stimulations, respectively, with *k*_CaN_ = 20 1/s. In all panels, the stochastic stimulation results are averaged over *N* = 300 synapses.

When the parameters of the model are chosen accordingly, the model reproduces qualitatively the experimental results in response to the STDP stimulation protocol: (i) short positive Δ *t* stimulation protocols move CaMKII from the weakly phosphorylated state to the highly phosphorylated state. Starting from the UP state, no transition occurs. (ii) A system at rest at the UP state is switched to the DOWN state by short negative Δ *t* protocols, whereas the same protocol does not evoke transitions from the DOWN to the UP state. (iii) Large positive and negative Δ *t*s do not evoke transitions between the DOWN and the UP states. We show in Figures 5–7 (red lines) the behavior of the model for parameters shown in Table 1A–1C, with *k*_CaN_ = 18 1/s and Δ*Ca*_pre_ = 0.17 μM.

Figure 5 shows the dynamics of the system for the whole stimulation protocol, and until the system has reached the final steady-state except for Figure 5A and 5B, which depicts the time course of the calcium concentration for one spike pair presentation only. Figure 5C and 5D shows the time course of active PP1, while Figure 5E and 5F shows the dynamics of phosphorylated CaMKII subunit concentration. The left column shows the dynamics of the system when it is initially in the DOWN state (low concentration of phosphorylated CaMKII subunits) for two representative time lags (Δ *t* = 15 ms and 100 ms), while the right column shows the dynamics when it is initially in the UP state, again for two representative time lags (Δ *t* = −50 and −10 ms).

To understand why the system exhibits transitions in specific ranges of Δ *t*, is it first crucial to examine how PP1 activation depends on Δ *t*. For the value of Δ*Ca*_pre_ chosen here, PP1 activation is the largest at short negative time differences, since such values of Δ *t* maximize the time spent by the system in the range of calcium concentrations close to PP1 peak activation (see Figure 3B). On the other hand, PP1 activation is minimal for short positive time lags since *Ca* goes transiently to high concentrations and spends a short time at intermediate values. Let us now focus on the situation in which the system is initially in the DOWN state. During the stimulation protocol, two situations can arise. For short positive time differences (as, for example, the 15 ms case shown in Figure 5), the increase in PP1 activity is low and insufficient to counterbalance the large increase in the concentration of phosphorylated CaMKII subunits, since high calcium transients strongly favor the autophosphorylation process which outweighs the low dephosphorylation activity. Hence, the system reaches a high phosphorylation level during the stimulation protocol and converges gradually toward its equilibrium value in the UP state thereafter. On the other hand, for negative and large positive time lags, the increase in PP1 activity is large enough to counterbalance the calcium/calmodulin triggered autophosphorylation, i.e*.*, CaMKII stays dephosphorylated and remains in the DOWN state (see for example the 100 ms case in Figure 5).

When the system is initially in the UP state, the concentration of phosphorylated CaMKII subunits again depends on the competition between dephosphorylation by PP1 and autophosphorylation progress during the protocol. Again, we have two possible outcomes of the protocol: either the PP1 concentration becomes large enough such that the system gets sufficiently dephosphorylated and moves in the basin of attraction of the DOWN state during the stimulation protocol (this occurs for example for the −10 ms case shown in Figure 5); or it is not large enough and autophosphorylation prevails, i.e*.*, the system remains in the basin of attraction of the UP state. For the parameter set used in Figure 5, this happens for large negative and positive time lags.

Another way of visualizing the dynamics during and after the STDP protocol consists in plotting the trajectory of the system in the concentration of phosphorylated CaMKII subunits *S*_active_–PP1 activity plane. This is done for several values of Δ *t* in Figure 6A and 6B. The DOWN and the UP stable steady-states of the CaMKII phosphorylation level are shown by the diamond and the cross, respectively (located at the intersections of the *S*_active_ and (*k*_12_ · D) nullclines). In Figure 6A the system is initially in the DOWN state, whereas it is initially in the UP state in Figure 6B. In both Figure 6A and 6B, the end of the stimulation protocol corresponds to the point at which PP1 and *S*_active_ stop to oscillate, and there is a sharp turn of the trajectories in the plane. In this plane, the separatrix (dotted black line) marks the boundary between the basins of attraction of both stable steady-states. Depending on the position of the system at the end of the stimulation protocol relative to this separatrix, the system relaxes either to the UP or the DOWN state. The separatrix is obtained by adjusting numerically Δ *t* to be at the boundary between the regions in which a transition to the UP (respectively, DOWN) state occurs or not.

The outcomes of the deterministic STDP protocols for Δ *t* values from −100 to 150 ms are summarized in Figure 7A (red line). We consider a large population of independent synapses submitted to the same protocol, in which initially half of the synapses are in the DOWN state and the other half in the UP state. Figure 7A shows the relative change in the fraction of synapses in the UP state as a function of Δ *t* (+1 means all synapses initially in DOWN where switched to UP; 0 means no change; −1 means all synapses in UP have switched to DOWN). There is a range of values of Δ *t* (from 10 to 16 ms) for which all synapses initially in the DOWN state switch to the UP state (LTP). LTD, or up-to-down transitions of the synapses initially in the UP state, is observed in a range of Δ *t* values from −14 to −2 ms (see red line in Figure 7A).

### STDP Protocol Stimulation with Stochastic Calcium Dynamics

The CaMKII kinase-phosphatase system in the PSD is composed of a few molecules only (**∼**30 CaMKII holoenzymes [56]), hence stochastic fluctuations potentially play an important role (see [57]). The CaMKII system is also exposed to fluctuating calcium transients stemming from stochastic neurotransmitter release, stochastic channel opening, and the stochastic nature of neurotransmitter as well as calcium diffusion [54,58]. It is therefore necessary to investigate the dynamic behavior of the CaMKII system in the presence of noise. Here we choose for simplicity to introduce fluctuations in calcium transients exclusively.

Two sources of noise are introduced in the calcium dynamics simulations: (i) the NMDA receptor maximum conductance is drawn at random at the occurrence of each presynaptic spike, and (ii) the maximum conductance of the voltage-dependent calcium channel is drawn at random at the occurrence of each postsynaptic spike. Both conductances are drawn from binomial distributions similar to those measured in experiments [58,59] (see Materials and Methods for more details). Some examples of noisy calcium transients are shown in the inset of Figure 7A (dashed lines; the full line depicts the average transient).

Again, we consider a large population of independent synapses exposed to stochastic stimulation protocols. *N* = 300 independent synapses are simulated, 150 initially in the DOWN and 150 in the UP state. Applying the stimulation protocol leads to stochastic transitions between UP and DOWN states. Figure 7A shows the relative change in the fraction of synapses in the UP state as a function of Δ *t*, for *k*_CaN_ = 18 1/s and 20 1/s. For example, a relative change of −0.8 for Δ *t* = −15 ms (*k*_CaN_ = 20 1/s case) means that 120 of the synapses in the UP state (out of the 150) switched to the DOWN state in response to this protocol, while none of the 150 synapses in the DOWN state experienced a down-to-up transition during the Δ *t* = −15 ms stimulation.

The variability in maximum NMDA and CaL current conductances results in a variability of calcium transients around the mean transients. The consequence of this variability in calcium transients is that, while the shape of the PP1 level versus Δ *t* is qualitatively unchanged, the PP1 level reached during stimulation protocols is significantly reduced. This is due to the fact that the variability in calcium transients decreases the time spent by the system at calcium concentrations which maximize PP1 buildup. Hence, the probability that the PP1 level is high enough to make the system switch to the DOWN state becomes small for *k*_CaN_ = 18 1/s (the value used in deterministic simulations). Consequently, the up-to-down transition probability at short negative time lags is low and LTD is effectively absent in this case (see green line with squares in Figure 7A). However, the LTD probability becomes larger as *k*_CaN_ is increased. Figure 7A shows an up-to-down switching probability of about 0.93 for short negative time lags with *k*_CaN_ = 20 1/s (at **Δ** *t* = −10 ms). It also shows that for this value of *k*_CaN_ there exists a small but finite probability of eliciting LTD transitions for large positive and large negative time lags, due to variability in calcium transients. The range of values of *k*_CaN_ for which the LTD probability for short negative Δ *t* is larger than 0.5 AND the LTD probability for large positive Δ *t* is smaller than 0.5 is 19 1/s < *k*_CaN_ < 20 1/s. To summarize, down-to-up transitions occur robustly for a large range of parameters at short positive time lags. On the contrary, the range of short negative values of Δ *t* for which UP to DOWN switches are observed is less robust to noise (see Discussion).

### Effect of Phosphatase Inhibitors

The role of protein phosphatases in synaptic plasticity has been investigated through the application of phosphatase inhibitors during the presentation of stimulation protocols inducing synaptic changes [21,37,43,60]. These experiments have shown that phosphatase inhibitors prevent LTD while sparing LTP. We investigate the effect of phosphatase inhibitors in our model by gradually reducing the dephosphorylation activity of PP1 and study the changes in the steady-states of the phosphorylated CaMKII subunit concentration and in the transition behavior.

**Figure 8:**
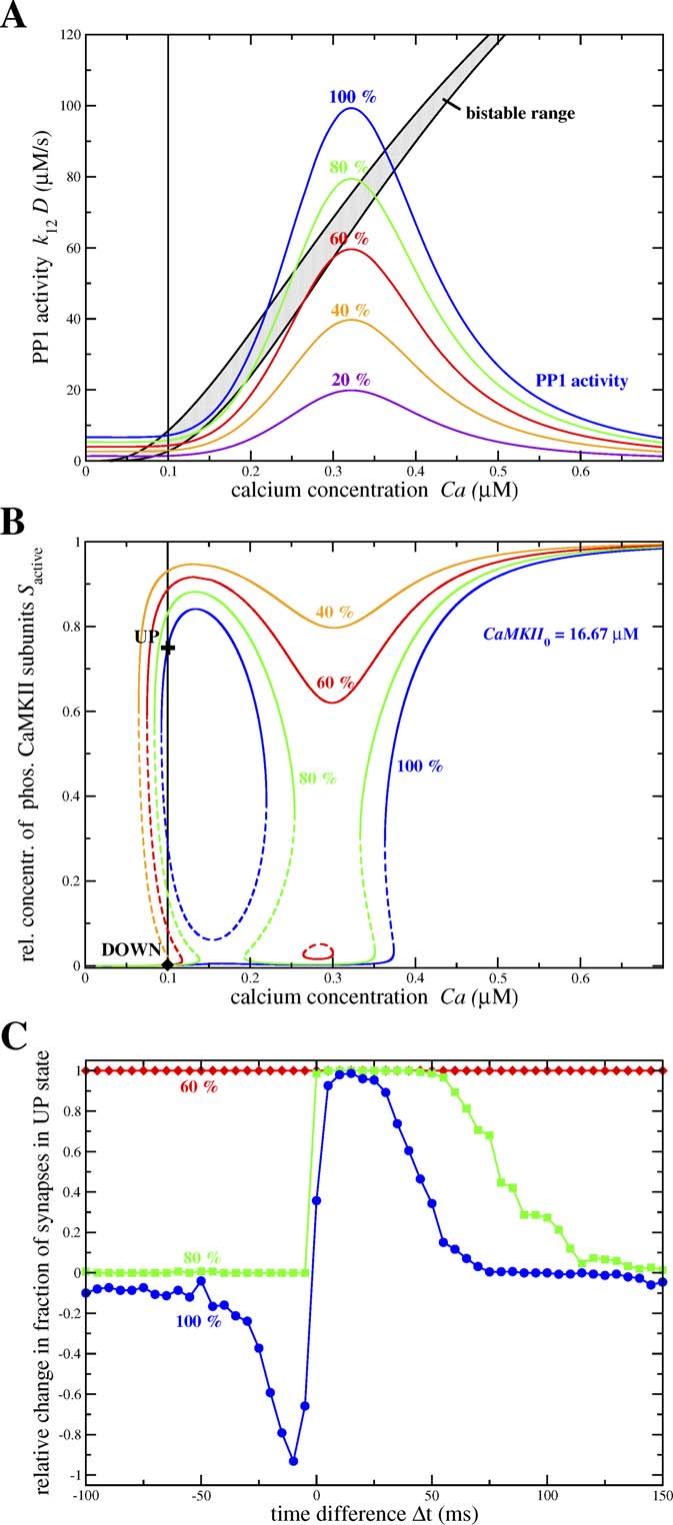
Phosphorylated CaMKII Subunit Concentration Steady-States and Transition Outcomes in the Presence of PP1 Inhibitors (A) PP1 activity as a function of calcium concentration, for different levels of inhibition (indicated close to the corresponding curves). For example, in the 80% case, the total PP1 concentration is 0.8 *D*_0_, i.e., 0.16 μM. The 100% curve corresponds to no PP1 inhibition (same curve as in Figure 3B). Vertical black line: calcium resting concentration, *Ca*_0_ = 0.1 μM. (B) Steady-states of the phosphorylated CaMKII subunit concentration versus Calcium for different levels of PP1 inhibition (same colors as in (A)). Full lines: stable steady-states. Dashed lines: unstable steady-states. The positions of the UP and the DOWN state at calcium resting conditions are shown for the 100% PP1 concentration case by the cross and the diamond, respectively. *CaMKII*_0_ = 16.67 μM, *k*_CaN_ = 18 1/s, see Table 1 for other parameters. (C) Transition results in response to the STDP stimulation protocol evoking noisy calcium transients in the presence of PP1 inhibitors. The average relative changes in the fraction of synapses in the UP state for three different total PP1 concentrations is shown (same colors as in (A) and (B)). The blue line is the same as the blue line in Figure 7A for *D*_0_ = 0.2 μM as given in Table 1, i.e*.*, the 100% case (*N* = 300, see Table 1 for other parameters).

Since the steady-state PP1 concentration is given by *D*_steady-state_ = *D*_0_ / (1 + (*I*_0_ *k*_13_ *v*_PKA_) / (*k*_-13_ *v*_CaN_)) (see Materials and Methods), scaling down *D*_0_ corresponds to a reduction of the steady-state PP1 activity given by the purple line in Figure 3B. Consequently, the intersections between the boundaries of the bistable region (given by the red lines in Figure 3B) and the PP1 activity change. In other words, the locations and ranges of the LTD and the LTD windows change as a function of the level of PP1 inhibition. Scaling down the total PP1 concentration leads to a diminution of the size of the LTD window and to the emergence of a second LTP window at low calcium concentrations (see the 80% case shown by the green line in Figure 8A and 8B). Decreasing further protein phosphatase strength makes the LTD window disappear and a large LTP window emerges starting at low calcium concentrations (see 60% and 40% cases in Figure 8A and 8B). Finally, reducing the PP1 concentration below ∼40% results in a loss of the stability of the DOWN state at resting calcium concentrations, leaving the UP state as the only stable steady-state for all calcium concentrations.

Figure 8C shows how LTP/LTD transitions are affected by reduced total PP1 concentration, when the model is exposed to the STDP stimulation protocol with noisy calcium transients. Consistent with experiments, reducing the PP1 concentration by 20% leads to a loss of LTD transitions (see green line in Figure 8C), while increasing the range of Δ *t* for which LTP transitions are observed. Further reduction of the PP1 concentration to 60% and more results in up-to-down transitions for all time differences Δ *t* (see red line in Figure 8C).

### Presynaptic or Postsynaptic Stimulation at Different Frequencies

Experiments on STDP show that presynaptic or postsynaptic spikes alone at the same stimulation frequency (1 Hz) do not evoke any plasticity (see for example [55]). To check the behavior of the model in this situation, we expose the CaMKII system to either 60 presynaptic or postsynaptic spikes of different frequencies and show the transitions results for the deterministic calcium transient case in Figure 7B by red lines. 60 presynaptic spikes alone do not evoke any transitions at low frequencies (1–3 Hz). For presynaptic stimulations in the range 4–18 Hz, up-to-down transitions occur, and for frequencies equal and larger to 19 Hz the CaMKII system is switched from the DOWN to the UP state (see full red line in Figure 7B). The transition outcomes change dramatically if stimulation occurs exclusively with postsynaptic spikes. 60 postsynaptic spikes do not evoke transitions up to a stimulation frequency of 84 Hz (see dashed red line in Figure 7B). Above 85 Hz, transitions from DOWN to UP occur. Note that the spike pairs during the STDP spike-pair stimulation protocol employed above are presented at a frequency of 1 Hz only, i.e., presynaptic or postsynaptic spikes alone at this frequency do not evoke transitions, consistent with experiments [55]. Note also that the model does not incorporate frequency-dependent attenuation of EPSPs and BPAPs. Attenuation of BPAPs at high frequencies could prohibit down-to-up transitions in the post protocol at any frequency.

We also expose the CaMKII system to fluctuating calcium transients evoked by presynaptic or postsynaptic frequency stimulations. The implementation of calcium transient noise is exactly as for STDP spike-pair protocols above. The average relative changes in the fraction of synapses in the UP state for these stimulations are shown for varying frequencies in Figure 7B for *k*_CaN_ = 18 1/s and 20 1/s (*N* = 300 synapses). Presynaptic stimulations at frequencies between ∼2 and ∼16 Hz evoke a net increase of synapses in the DOWN state, while stimulation above ∼16 Hz lead to LTP transitions. Again, no up-to-down transitions are observed with postsynaptic stimulation alone, while stimulation frequencies above ∼50 Hz yield a net increase of the number of synapses in the UP state.

### Presynaptic or Postsynaptic Spike-Pair Stimulation at Different Time Differences

Another simple generalization of the STDP protocol consists in exposing the system to purely presynaptic spike pairs, or purely postsynaptic spike pairs. Spike pairs with a fixed inter-spike interval Δ *t* are presented 60 times at varying frequencies. We investigate the transition behavior of the model for varying inter-spike intervals and for different presentation frequencies. This is a protocol for which plasticity outcomes have, to our knowledge, not yet been characterized.

Presynaptic spike pairs lead to up-to-down transitions for all values of Δ *t* at a frequency of *f* = 1 Hz, consistent with the fact that presynaptic stimulation of single spikes at 2 Hz evokes such transitions (see Figure 7B). On the other hand, purely postsynaptic spike pairs evoke down-to-up transitions in a very narrow range of values of Δ *t* (from 3 to 8 ms) at *f* = 1 Hz. In other words, postsynaptic spike pairs have to be presented sufficiently closely in time for the phosphorylation changes to sum up, so that the system converges to the UP state. Decreasing the spike-pair presentation frequency *f* leads to transitions in narrower ranges of Δ *t* for the presynaptic and the postsynaptic protocol (e.g., *f* = 0.5 Hz; presynaptic spike pairs with 0 < Δ *t* ≲ 300 ms lead to up-to-down transitions, postsynaptic spike pairs with 3 ≲ Δ *t* ≲ 6 ms evoke down-to-up transitions). At *f* = 0.1 Hz, there are no longer any transitions in the purely presynaptic stimulation protocol and only a small down-to-up transition probability exists for postsynaptic spike pairs (3 ≲ Δ *t* ≲ 4 ms; unpublished data).

The difference in transition outcomes between presynaptic and postsynaptic spike-pair stimulations can be understood by inspecting the calcium transients evoked by both stimulation protocols. The maximum calcium amplitude reached by pairs of postsynaptic spikes is much larger than the calcium amplitude evoked by presynaptic spikes (maximum amplitude for Δ *t* = 10 ms is ∼0.45 μM for presynaptic spike pairs and ∼0.7 μM for postsynaptic spike pairs with the parameters given in Table 2). On the other hand, pairs of presynaptic spikes evoke calcium transients which last much longer than postsynaptic pairs of spikes (compare the different time scales in Figure 4). The high calcium transients evoked by postsynaptic spike pairs strongly activate the cAMP–PKA pathway and therefore suppress PP1 activity. This suppression, together with the strong CaMKII autophosphorylation due to high calcium concentrations, leads to down-to-up transitions in response to closely spaced postsynaptic spike pairs. Even single postsynaptically evoked calcium transients reach calcium levels sufficiently high to activate the cAMP–PKA pathway. This explains why purely postsynaptic stimulation at varying frequencies does not go through an LTD range (see Figure 7B and compare the small PP1 buildup in the inset in Figure 5D in response to the postsynaptically evoked calcium transient in the Δ *t* = −50 ms protocol). In contrast, the long-lasting calcium transients evoked by purely presynaptic spike pairs make the system spend a lot of time in calcium ranges maximizing PP1 buildup. This leads to strong dephosphorylation of CaMKII by PP1 which cannot be counterbalanced by moderate autophosphorylation evoked by intermediate calcium levels. Hence up-to-down transitions are evoked for closely spaced presynaptic spike pairs. Again, this explains also why ongoing presynaptic stimulation at different frequencies evokes LTD at low presentation frequencies before the calcium transients are adding up sufficiently to activate the cAMP–PKA pathway and evoke strong autophosphorylation (this happens above *f* ≈ 16 Hz in Figure 7B).

### Effect of Parameter Changes on the Behavior of the Model

The model with the parameter set discussed until this point reproduces qualitatively experimentally observed transition outcomes of the STDP protocol. We now discuss how changing parameters affect the transition behavior. Numerical investigations of the model show that two characteristics of the CaMKII system dynamics are crucial: (i) how the level of PP1 activity at the end of the stimulation protocol depends on Δ *t*; (ii) the time course of autophosphorylation and dephosphorylation of CaMKII, and of PP1 buildup, influences the number of spike pairs during the stimulation protocol necessary to evoke down-to-up- or up-to-down transitions. We focus here for the sake of simplicity on deterministic calcium transients.

### Effect of the Amplitude of Calcium Transients and of Calcineurin Activity on the PP1 Activity Level

**Figure 9:**
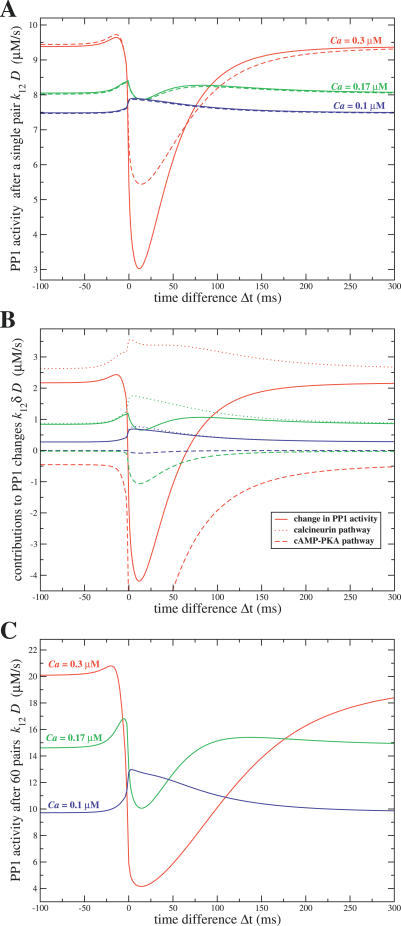
PP1 Activity Level After the Presentation of One or 60 Spike Pairs as a Function of Δt and the Amplitude of Calcium Transients (A) PP1 level after the presentation of one spike pair as a function of Δ *t*. Dashed lines: numerical integration of Equation 24 and 25. Full lines: approximate solution (*k*_12_ (*D* ^*^ + *δD*(*t*))) from numerical integration of Equation 34. The three different colors correspond to three different calcium amplitudes Δ*Ca*_pre_ as marked in the panel. (B) The increase or decrease of PP1 activity induced by a single spike pair (full lines) as well as the contributions from the calcineurin pathway (dotted lines) and the cAMP–PKA pathway (dashed lines) to this change in PP1 activity as a function of Δ *t*: the three colors correspond to the same three different calcium amplitudes Δ*Ca*_pre_ as marked in (A). The dotted and dashed lines are obtained from numerical integration of the first and the second terms in Equation 34, respectively. The full lines depict the sum of both contributions and represent *k*_12_ · *δD*(*t*) after the presentation of the first pair of spikes (see Materials and Methods). (C) Total level of PP1 activity after the *whole* stimulation protocol from numerical integration of Equations 24 and 25, as a function of Δ *t*: The different colors correspond again to the three different calcium amplitudes as in (A) and (B) (see Table 1 for parameters, *k*_CaN_ = 18 1/s).

We have shown above that a peak in steady-state PP1 activity at moderate calcium concentrations occurs if the cAMP–PKA pathway activates at higher calcium concentrations than the calcineurin pathway (see Figure 3A). Here, we address the question of how the dynamics of the PP1 activity during the stimulation protocol changes as a function of the balance between the activation of both pathways. Changing the NMDA-R mediated calcium amplitude Δ*Ca*_pre_ and the BPAP evoked calcium response, keeping their ratio constant (Δ*Ca*_post_ = 2 · Δ*Ca*_pre_ ), and also keeping the parameters of the protein signaling cascade constant, allows us to change the balance between the activation of both pathways and to get an insight into what controls the dependence of the PP1 activity level on Δ *t*. Figure 9A and 9C show the PP1 activity level immediately after the presentation of *one* and *60* spike pairs, respectively, as a function of Δ *t*, for different values of Δ*Ca*_pre_. Note that the dependence of PP1 activity with respect to Δ *t* after the presentation of one spike pair (Figure 9A) is qualitatively preserved after the entire stimulation protocol of 60 spike-pair presentations (Figure 9C).

Figure 9B represents the change in PP1 activity induced by a single spike pair, computed from Equation 34, as well as contributions of the PKA and calcineurin pathways to this change. The dashed lines in Figure 9B show the contribution of the cAMP–PKA pathway to the change in PP1 activity (second term in the integral of Equation 34). This contribution is negative, since this pathway decreases PP1 activity. Due to the high half activation calcium concentration of *v*_PKA_(*C*) (see blue line in Figure 3A), the cAMP–PKA pathway is sensitive to high calcium elevations only. Hence, the negative contribution of this pathway increases drastically when the calcium amplitude Δ*Ca*_pre_ increases, since the calcium transients spend more time in the range of cAMP–PKA activation. In response to the supralinear superposition of the NMDA-R and the BPAP evoked currents at short positive time differences, this pathway ensures a low level of PP1 activity in this range.

The dotted lines in Figure 9B show the contribution of the calcineurin pathway to the change in PP1 activity. This contribution is positive, since this pathway increases PP1 activity. The calcineurin pathway activates at lower calcium concentrations than the PKA pathway (see red line in Figure 3A; integral of the first part of Equation 34), and therefore this pathway is sensitive to the time spent by the system at intermediate and high calcium levels. This calcineurin contribution starts to increase at negative time differences (when calcium transients induced by pre- and post-synaptic spikes start to interact), reaches a peak close to Δ *t* = 0, and then decreases slowly with Δ *t*.

The sum of the two contributions yields the net change in PP1 activity (full lines of Figure 9B). For Δ*Ca*_pre_ = 0.17 μM, the value chosen in the rest of the paper, the PP1 change versus Δ *t* curve shows first a peak at negative Δ *t* (due to increase in calcineurin activity in this range), followed by a trough at positive Δ *t* (due to the strong increase in PKA activity in this range). There is a secondary peak of PP1 change at larger values of Δ *t* (∼100 ms) because calcineurin activity decays more slowly with Δ *t* than PKA activity. However, this peak is smaller than the peak at negative Δ *t*, which explains why LTD is observed at short negative Δ *t* but not large positive ones.

Changing the size of the calcium transients potentially changes qualitatively the shape of this curve because it affects the time spent by the system in different calcium concentration ranges. For example, decreasing the size of the calcium transients weakens considerably the PKA pathway, leading to an increase in PP1 activity for negative as well as positive values of Δ *t*. On the other hand, increasing the calcium transients leads to a strengthening of the PKA pathway relative to the calcineurin pathway, leading to a much smaller peak in the PP1 change curve at short negative Δ *t*. This peak eventually vanishes for large enough Δ*Ca*_pre_ ≥ 0.4 μM (unpublished data).

Since transitions are a result of an unbalance between autophosphorylation and dephosphorylation mediated by PP1, the Δ *t* range for which transitions are evoked or prevented can therefore be controlled by means of the calcium amplitude. If the calcium amplitude is decreased in the model, no transitions are observed any more (e.g., for Δ*Ca*_pre_ = 0.15 μM). On the other hand, increasing the calcium amplitude extends the Δ *t* range for which up-to-down and down-to-up transitions are evoked (Δ*Ca*_pre_ = 0.18 μM, LTD range: [−21... −3] ms and LTP range: [3...33] ms; unpublished data). These predictions could be checked experimentally by changing the external calcium concentration and therefore changing the calcium influx evoked by presynaptic and postsynaptic spikes.

To summarize, there exists a range of Δ*Ca*_pre_ for which the PP1 level at the end of the stimulation protocol as a function of Δ *t* exhibits a maximum for short negative Δ *t*s and is low enough to be outweighed by autophosphorylation for short positive Δ *t*s. This is a requirement for a system to exhibit LTD-like transitions at short negative time intervals only, *and* LTP-like transitions at short positive time intervals only. However, these qualitative features of the PP1 activation versus Δ *t* curve are not sufficient to ensure that STDP protocol stimulations with short negative time lags lead to transitions from the UP to the DOWN state only. In addition, (i) the absolute level of PP1 activity for short negative Δ *t* stimulations must be high enough to evoke up-to-down transitions; (ii) at the same time, the total PP1 level has to be low enough such that for large negative and large positive time lag stimulations the system remains in the UP state and that for short positive Δ *t* protocols autophosphorylation prevails over dephosphorylation leading to down-to-up transitions. These two criteria can be met by changing the maximal calcium/calmodulin-dependent calcineurin activity *k*_CaN_, which changes the amplitude of the peak of the PP1 vs Ca^2+^ steady-state curve at moderate calcium concentrations (purple lines in Figure 3B). Consequently, this parameter allows us to control the PP1 level attained during the stimulation protocol for all **Δ** *t*s. In particular, the range 16.6 ≤ *k*_CaN_ ≤ 18.1 1/s fulfills the two requirements above (Figures 5 and 6 and the red as well as the green lines in Figure 7 use *k*_CaN_ = 18 1/s).

### Effect of Kinetics of Autophosphorylation and Dephosphorylation on the Number of Spike-Pair Presentations Needed for Transitions

The autophosphorylation rates *k*_6_, *k*_7_, *k*_8_, the maximal dephosphorylation rate *k_12_*, and the total PP1 concentration *D*_0_ determine the velocity of autophosphorylation as well as dephosphorylation dynamics of CaMKII and the dynamics of the PP1 response during exposure to the STDP protocol. We introduce scaling parameters *R* and *Q* such that varying *R* and *Q* does not change the steady-state behavior of the CaMKII system (see Figure 3B and 3C) nor the maximum PP1 activity reached during the stimulation but only the dynamics of the system. Both scaling parameters are varied extensively in order to investigate their impact on the transition behavior of the model, i.e*.*, 0.002 ≤ *R* ≤ 2 and 0.083 ≤ *Q* ≤ 1.67.

**Figure 10:**
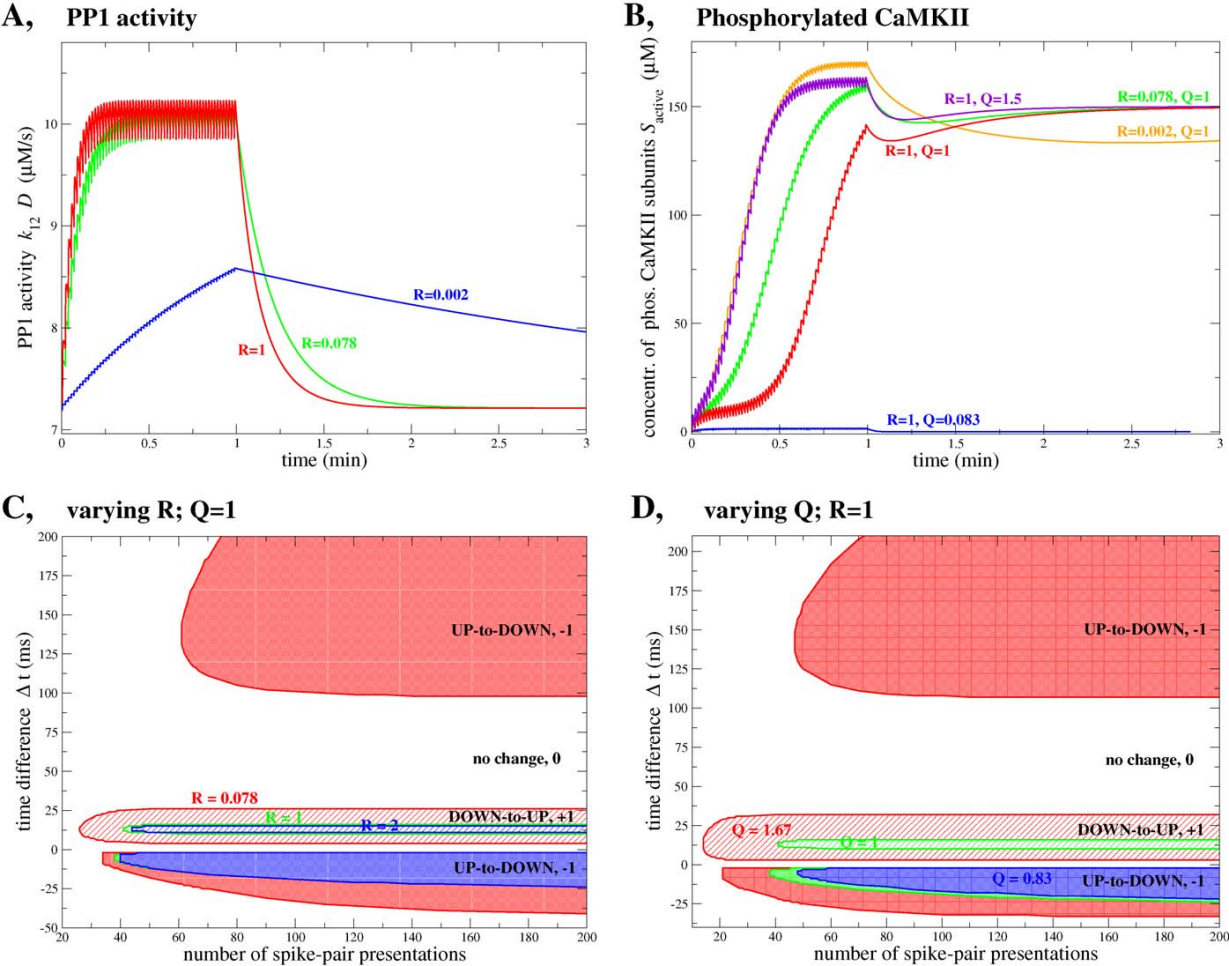
Impact of the Kinetics of PP1 Activity and CaMKII Phosphorylation on the Number of Spike-Pair Presentations Leading to Transitions (A,B) Time course of PP1 activity (*k*_12_ · *D*) (A) and phosphorylated CaMKII subunit concentration (B) during and after the STDP stimulation protocol for different values of *R* and *Q* (deterministic stimulation protocol, 60 spike pairs with Δ *t* = 15 ms). The buildup of PP1 activity (*k*_12_ · *D*) is shown in (A) for *R* = 0.002 (blue line), *R* = 0.078 (green line) and *R* = 1 (red line; same curve as in Figure 5C). *Q* has no impact on PP1 dynamics. The time course of phosphorylated CaMKII subunit concentration is depicted in (B) for five sets of values of *R* and *Q* (indicated close to the corresponding curves). (C,D) The impact of the number of spike-pair presentations and of *R* and *Q* on the Δ *t* ranges for which transitions occur is depicted for STDP stimulation protocols evoking deterministic calcium transients. White region: no change (relative change in fraction of synapses in the UP state is 0); diagonally striped regions: down-to-up transitions (relative change in fraction of synapses in the UP state is +1); shaded regions: up-to-down transitions (relative change in fraction of synapses in the UP state is −1). In each case, down-to-up and up-to-down transition regions in the same color correspond to the same choice of *R* (C) or *Q* (D). All the cases in (C) use *Q* = 1 paired with: *R* = 0.078: red regions; *R* = 1: green regions; and *R* = 2: blue region. In (D): blue regions: *Q* = 0.83 (no down-to-up transitions); green regions: *Q* = 1; and red regions: *Q* = 1.67. *R* = 1 is utilized in all cases in (D), i.e., the green regions in (C) and (D) are identical (see Table 1 for parameters, *k*_CaN_ = 18 1/s, Δ*Ca*_pre_ = 0.17 μM).

*R* is chosen such as to control the dephosphorylation kinetics, while leaving the PP1 activity, given by the product (*k_12_* · *D*), constant. This leaves the steady-state behavior intact since it depends on this product only. Hence, in the following simulations, *k*_12_ and *D*_0_ are replaced by 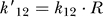 and 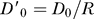 , where *k*_12_ and *D*_0_ are the “control” parameters listed in Table 1B. *R* controls how fast the dephosphorylation dynamics responds to calcium transients, since the PP1 buildup during the presentation of the stimulation protocol and the decay dynamics thereafter depend on the value of *D* but not on *k*_12_ (see Equation 31 in Materials and Methods). Figure 10A shows the PP1 activity time course for three different values of *R* during and after the STDP stimulation protocol with Δ *t* = 15 ms, i.e., for *R* = 0.002, 0.078, and 1.

*Q* scales the autophosphorylation rates *k*_6_, *k*_7_, and *k*_8_ together with the maximal dephosphorylation rate *k*_12_ as 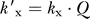 (with x = 6,7,8, and 12), where all the rates *k_x_* take the values listed in Table 1B. This corresponds to a rescaling of the *y*-axis in Figure 3B, i.e., the points of intersection between the bistable range (red shaded areas) and the PP1 activity (purple lines) are kept fixed and therefore the steady-state concentration of phosphorylated CaMKII subunits (Figure 3C) is left unchanged. We illustrate the impact of changes in *Q* on the dynamics of *S*_active_ in Figure 10B for three different *Q*s and *R* = 1 as well as three different *R*s and *Q* = 1. Since the temporal evolution of *S*_active_ is a result of the competing autophosphorylation and dephosphorylation progress, the choice of *both* scaling parameters influences *S*_active_ dynamics (see Figure 10B). Note that *R* = 1 and *Q* = 1 is used everywhere in this paper, except for the results discussed in this section and shown in Figure 10.

Increasing *R* accelerates the convergence of PP1 toward a steady-state oscillation. In Figure 10A, this happens after ∼20 and ∼30 spike-pair presentations for *R* = 1 and *R* = 0.078, respectively. This constant value is not attained during the 60 s stimulation protocol with *R* = 0.002 at all. Reaching such a steady-state behavior is needed for the system to be robust to changes in the number of spike-pair presentations. Indeed, if the PP1 activity is still in the raising phase at the end of the stimulation protocol (as in the case *R* = 0.002, see blue line in Figure 10A), then more spike-pair presentations would lead to a higher PP1 level and therefore to up-to-down transitions for a drastically wider range of Δ *t* values if the system is initially in the UP state. On the other hand, less spike-pair presentations would not give rise to any transitions at all. Figure 10C and 10D give an insight on how the Δ *t* range for which transitions occur depends on *R*, *Q*, and the number of spike-pair presentations. For *R* = 1 and *Q* = 1 (*k*_CaN_ = 18 1/s, Δ*Ca*_pre_ = 0.17 μM), the range of Δ *t* values evoking down-to-up transitions saturates beyond 50 spike-pair presentations, whereas the range resulting in up-to-down transitions becomes essentially insensitive to the number of spike-pair presentations beyond ∼150 spike pairs (see green regions in Figure 10C and 10D; both depict the same results). Increasing *R* further does not lead to any significant changes in the ranges of transitions compared to the case *R* = 1 (compare green and blue regions in Figure 10C for *R* = 1 and *R* = 2, respectively, and see Materials and Methods). Decreasing *R* slows down the convergence toward a stable range of time lags evoking down-to-up or up-to-down transitions (compare *R* = 1, green regions, and *R* = 0.078, red regions, cases in Figure 10C). This is due to the slower convergence of PP1 activity to its oscillatory behavior around a constant value during the stimulation protocol (see Figure 10A). The examples in Figure 10A show furthermore that the smaller *R*, the slower the decay of PP1 activity after the stimulation protocol. When *R* = 0.078 the PP1 dephosphorylation activity decay after the stimulation protocol is so slow that large positive time lag stimulations evoke transitions from UP to DOWN (see upper red shaded region in Figure 10C, *Q* = 1, *k*_CaN_ = 18 1/s, Δ*Ca*_pre_ = 0.17 μM). Up-to-down transitions at large positive time lags appear in the range up until 200 spike-pair presentations for *R* ≲ 0.202.

Similar arguments hold for the scaling parameter *Q* that controls CaMKII autophosphorylation and dephosphorylation dynamics. Indeed, the degree of CaMKII subunit phosphorylation should also reach a steady oscillation around a constant value during the presentation of spike pairs, for the system to exhibit robust behavior (see the *R* = 1,*Q* = 1.5 and *R* = 1,*Q* = 0.083 cases in Figure 10B). The larger the *Q*, the faster autophosphorylation (through an increase of *k*_6_, *k*_7_, and *k*_8_; Figure 1C–1E) and dephosphorylation (through an increase of *k*_12_, Equation 6) proceed. Therefore, less spike-pair presentations are required to evoke transitions and the Δ *t* ranges leading to down-to-up- or up-to-down transitions saturate at smaller numbers of spike-pair presentations (see Figure 10D). For *Q* = 0.083, no transitions are observed at all in the range from 1 to 200 spike-pair presentations (see blue line in Figure 10B). Down-to-up transitions appear after 60 spike-pair presentations for 1≲ *Q* (*k*_CaN_ = 18 1/s, *R* = 1) whereas up-to-down transitions occur at lower *Q* values (see (*R* = 1,*Q* = 0.83) case in Figure 10D). For 1.25 ≲ *Q*, dephosphorylation progress wins over autophosphorylation and leads to up-to-down transitions for large positive time lags (see red regions for *R* = 1,*Q* = 1.67 in Figure 10D).

## Discussion

### Bistability

The minimal stimulation protocols of Petersen et al. and O'Connor et al. on single hippocampal CA3-CA1 synapses evoke step-like all-or-none transitions of synaptic transmission efficacy [20,21]. These experiments suggest that individual synapses store information in a digital manner. The model presented here exhibits two stable steady-states of the CaMKII phosphorylation level at resting calcium conditions. This idea dates back from the pioneering paper by Lisman (1985) [15] and has since been investigated by modeling studies of increasing biochemical realism [15,17,18,42,57,61]. Miller et al*.* showed that the highly phosphorylated state in a system composed of a realistic number of CaMKII holoenzymes can remain stable on very long time scales (years) in the presence of protein turnover [57]. Hayer and Bhalla found that CaMKII bistability is preserved in the context of translocation and localization in the PSD of the protein [42]. The UP state in their study possesses a predicted lifetime of the order of tens of hours which is supported by experimental studies reporting a prolonged localization of the CaMKII at its postsynaptic site following LTP stimulation [25,62,63].

Experimentalists have reported several types of synaptic increase or decrease. For example, LTD (decrease of efficacy from “basal” strength) and depotentiation (decrease of efficacy after potentiation) have often been considered as two distinct processes. Some of the differences between the two can be reconciled in our model by considering that a “basal” condition is likely to be a mix of synapses in the UP and the DOWN state. Hence, a LTD protocol will decrease synaptic strength by provoking up-to-down transitions in some synapses that were initially in the UP state. On the other hand, in depotentiation protocols the initial conditions are different, because a larger fraction of the synapses are in the UP state. However, some studies indicate that depotentiation and LTD might operate through different molecular mechanisms [64–66]. A more complex model than the one proposed here would be necessary to account for these experimental data.

### LTP/LTD Transitions at Fixed Calcium Concentration

As in previous models, our model exhibits LTP for high enough calcium concentrations. Unlike previous models however, it possesses an “LTD window”, where the system makes a transition from the highly phosphorylated to the weakly phosphorylated state, under plausible conditions. There are three requirements for LTP and LTD transitions to occur at realistic calcium concentrations in our model (see Figure 3C).

(I) The steady-state concentration of phosphorylated CaMKII subunits has to exhibit a bistable behavior, i.e., a highly and a weakly phosphorylated state should coexist in a range of calcium. This is the case if phosphatase activity saturates at high CaMKII phosphorylation levels. In turn, this is ensured if the phosphatase is present in small amounts compared to CaMKII, which itself is enriched at high concentrations in the PSD [24,27,47]. We show that bistability is a property of the CaMKII system which is robust to variations in the number of interacting subunits but most effectively expressed in a ring of biological size with six interacting subunits (see Figure 2A).

(II) The phosphatase activity at resting conditions has to allow for two stable CaMKII phosphorylation states. Bistability at *Ca*_0_ is a robust property of the model over a large range of values of most of the protein signaling cascade parameters (see Table 1B). However, this requirement constrains the calcium-independent activities of the calcineurin and the cAMP–PKA pathways and is the reason why the model presented here is sensitive to changes in PKA base activity 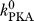 , i.e., varying 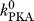 more than 14% from the value given in Table 1B leads to a loss of bistability at resting conditions.

(III) The “LTD window” emerges from an elevated phosphatase activity in the range of intermediate calcium concentrations. There are two possible realizations of the cAMP–PKA pathway for such an “LTD window” to arise: (i) if the PKA activity is assumed to be calcium-independent, the PP1 activity curve (purple lines in Figure 3B) would show a Hill-function–like behavior. However, a CaMKII versus calcium bifurcation diagram qualitatively similar to Figure 3C could still be obtained. How such a scenario would affect the behavior of the system in response to the STDP protocol is still to be clarified. (ii) If the cAMP–PKA pathway is calcium/calmodulin-dependent as chosen here (see also [61]), the PP1 activity can be coupled to the calcium concentration such that a peak emerges at intermediate calcium concentrations. Several lines of experimental evidence support the inclusion of such a calcium-dependent cAMP–PKA pathway which promotes LTP by blocking phosphatases in the model: the induction of hippocampal LTP is blocked by inhibiting cAMP-dependent protein kinase A or inhibition of postsynaptic kinases in general and is facilitated in a PKA-dependent manner by inhibiting calcineurin [38,60,67]; a rapid increase in PKA activity accompanies the early phase of LTP in afferent fibers between hippocampus and prefrontal cortex [68]; calcium-stimulable forms of cAMP exist which indirectly control PKA activity [69]. For the CaMKII system to exhibit the “LTD window” with a calcium/calmodulin-dependent cAMP–PKA pathway, the model predicts that the cAMP–PKA pathway should activate at higher calcium concentrations compared to the calcineurin pathway, as this is required for the peak of phosphatase activity to emerge.

Another way to assess the coupling of the protein signaling cascades to PP1 activity and to CaMKII is to check what the model predicts if we block different parts of the pathways and compare it to experimental results. We can implement the blockade of the calcineurin or the cAMP–PKA pathways in the model by removing the calcium/calmodulin-dependence of the calcineurin or the cAMP–PKA pathways, since inhibitor 1 is also dephosphorylated by the calcium-independent protein phosphatase 2A [70,71] and phosphorylated by the calcium-independent protein kinase G [72]. Blocking the calcium/calmodulin-dependent part of the calcineurin pathway (i.e*., k*_CaN_ = 0) leads to facilitation of LTP, and the reverse transition (LTD) is prevented. On the contrary, blocking the calcium/calmodulin-dependent part of the PKA pathway (i.e., *k*_PKA_ = 0) facilitates LTD and prevents LTP. Transitions in either one of both directions can be evoked since bistability at resting conditions is preserved in both cases. All these model predictions are consistent with experimental assays inhibiting either the calcineurin [37,43,60] or the cAMP–PKA pathway [38]. If either the calcineurin or the cAMP–PKA pathways are completely abolished in the model, i.e., both the calcium-independent and the calcium-dependent parts are suppressed (i.e., 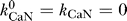 or 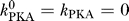 ), the system becomes locked in the UP or the DOWN state, respectively. Under these conditions, bistability is not present at resting calcium concentrations, i.e., no transitions can be evoked in a stable fashion. This also means a change in basal synaptic transmission since all synapses in the system will converge to one of the two stable states. Along the lines of the argumentation above, this situation would correspond to a scenario in which all proteins de- or phosphorylating inhibitor 1 are inhibited. Inhibiting completely protein phosphatase 1 activity, i.e., setting PP1 activity to zero, results in locking the system to the UP state for all calcium concentrations in our model. However, other calcium-independent phosphatases such as protein phosphatase 2A and 2C are known to dephosphorylate CaMKII [73]. Adding such phosphatases to the model would lead to bistability even in the absence of PP1. Such a scenario would be consistent with experiments which have shown that LTD but not LTP requires the activation of PP1 [37,39,74]. Our model indeed predicts a progressive diminution of the LTD window and an enlargement of the LTP window as a function of PP1 inhibition. In response to the STDP protocol, LTD disappears first when phosphatase activity is decreased as suggested by experimental results [67]. Reducing the phosphatase activity further results in down-to-up transitions for all Δ *t*s before the stable DOWN state disappears if the total PP1 concentration is reduced below 40%.

In addition to the “LTD window” at intermediate calcium concentrations, our model possesses a second region of bistability between the “LTD window” and the “LTP window” (see region between points **3** and **4** in Figure 3C). This region is not present in previous models and can be seen as a region of no changes. Starting from the DOWN or the UP state, calcium elevations to this range do not evoke any transition. A similar region of calcium concentrations in between LTP and LTD calcium levels leading to no plasticity is found experimentally by Cho et al. and discussed by Lisman as “no man's land” [75,76].

### LTP/LTD Transitions in Response to STDP Protocols

We have shown that the model can qualitatively reproduce plasticity outcomes in response to the STDP protocol. In our model as in previous models [9,10,77,78], the only signal driving synaptic changes is the dynamics of the calcium concentration, consistent with current experimental data [3,55,79–82]. However, previous modeling studies that use either the maximum amplitude of the calcium signal or simple readout mechanisms of the entire calcium dynamics reproduce only partially STDP results [9,10,77,78]. In particular, it has proven difficult to prevent the appearance of a second LTD range at large positive Δ *t*s. Shouval and Kalantzis show that stochastic properties of synaptic transmission can markedly reduce the LTD magnitude in this range [83]. Karmarkar et al. hypothesize that two functionally distinct calcium pools trigger different readout mechanisms for LTP and LTD in order to overcome this difficulty [9]. Here, we show that the compound calcium signal from VDCCs and NMDA-Rs combined with a complex readout mechanism is sufficient to account for experimental STDP data; in other words, the two calcium influxes do not have to be separated. This is due to the highly cooperative CaMKII autophosphorylation and the protein signaling cascade influencing PP1 activity, which provide a strongly nonlinear detector system, which is sensitive enough to translate differences in the time course of the calcium concentration into observed plasticity outcomes. Finally, CaMKII phosphorylation level changes need to sum over several pairs of spikes in order to observe LTP- or LTD-like transitions, as suggested by experiments on STDP [4,43,84–86]. These changes combine in a highly nonlinear fashion in our model, going beyond simple summation of pairwise interactions. In particular, a minimal number of spike pairs is needed to observe any plasticity, as shown in Figure 10. This number depends on the kinetics of autophosphorylation and dephosphorylation dynamics in the model. Froemke et al. (visual cortex slices) and Wittenberg and Wang (hippocampal slices) showed that LTP (causal spike pairings) requires only a few spike-pair presentations whereas the appearance of LTD (anti-causal pairings) requires a longer period of stimulation (∼100 spike pairings) [87,88]. Figure 10C and 10D show the faster saturation of the time lag range evoking LTP compared to the one evoking LTD, consistent with those experimental results. Interestingly, Wittenberg and Wang see a second LTD range at large positive time differences emerging after sufficiently long stimulation (70–100 spike pairings; compare second LTD window at positive Δ *t* emerging at high spike-pair presentation numbers in Figure 10C and 10D for (*R* = 0.078, *Q* = 1) and (*R* = 1, *Q* = 1.67), respectively).

Rubin et al. recently proposed a model based on pathways resembling the CaMKII kinase-phosphatase system, which reproduces experimental STDP outcomes but does not exhibit bistability [44]. In that model, high, short-lasting calcium levels evoke LTP, low and prolonged calcium elevations above a certain threshold evoke LTD, and intermediate calcium levels act like a “Veto” preventing LTD induction. The durations for which their detector system has to be exposed to respective calcium levels are consistent with our findings. The competition between the PP1 buildup level and the autophosphorylation progress implements naturally the concept of the veto in our model. This balance between PP1 activity and autophosphorylation changes with Δ *t* and defines the transition outcome: (short negative Δ *t*s) high PP1 accumulation and intermediate autophosphorylation of CaMKII evoked by linear interactions of the calcium influxes lead to LTD; (short positive Δ *t*s) low PP1 activity together with strong autophosphorylation of CaMKII as a result of supralinear calcium summations produce LTP; (all other cases) intermediate PP1 concentrations and weak to intermediate autophosphorylation arouse no changes. In particular, the stronger cAMP–PKA pathway activation due to higher calcium elevations for large positive Δ *t* protocols can be seen as a realistic veto preventing LTD transitions to occur in this range. The differential activation of competing pathways at different calcium levels receives further support by recent experimental studies [89].

We observe a larger extent of the range of Δ *t* values evoking LTD compared to the LTP range for *R* = 1 and *Q* = 1 in the noiseless case (see Figure 10D, green regions). In our model, the LTD range can be either larger, or smaller, than the LTP range, depending on various parameters such as noise, *R*, and *Q*. For large noise levels, the LTD range is generally smaller than the LTP range, while experimental data seems to indicate the opposite trend (compare blue line in Figure 7A and [2,4,85]). Investigating extensively how the parameters of the system change the extent of the LTP and LTD ranges goes beyond the scope of this study. In any case, the range of Δ *t*s leading to up-to-down transitions cannot be extended beyond the range of interaction between both calcium influxes. Hence, we expect the LTD range to become larger if this interaction is extended, e.g., due to nonlinear buffer dynamics [44] or the recruitment of additional protein signaling cascades [55,75,90]. Furthermore, BPAP attenuation and broadening has been shown experimentally to affect the STDP results and could change the balance between the ranges of time lags evoking LTP and LTD in our model [87,91]. Our model predicts that the range of time lags evoking LTP in response to the STDP protocol can be increased by amplifying PKA activity. On the other hand, increasing the strength of the calcineurin pathway shifts down horizontally the entire STDP curve (Figure 7A), leading to LTD transitions at all Δ *t*s (unpublished data).

Our model also reproduces qualitatively experimental transition results evoked by a purely presynaptic stimulation protocol [3]. Low stimulation frequencies evoke LTD and high frequencies LTP with a transition from LTD to LTP at 16–17 Hz in our simulations (compare with [3] where the transition happens at around 10 Hz but 900 presynaptic spikes are presented, instead of 60 here), for the same parameters that fit qualitatively the STDP data. Our model furthermore predicts that postsynaptic frequency stimulation evokes LTP at frequencies above 50 Hz (see Figure 7B). Interestingly, this type of stimulation does not evoke transitions from UP to DOWN at any frequency. However, we expect this form of plasticity to be strongly dependent on the extent and the time course of BPAP amplitude suppression. We also exposed the CaMKII system to purely presynaptic or postsynaptic spike-pair stimulation protocols. Since presynaptic spikes evoke long-lasting calcium transients and postsynaptic spikes high but fast-decaying calcium elevations, the Δ *t* ranges for which transitions can be observed in the two cases are markedly different. In particular, our model predicts pairs of postsynaptic spikes should elicit down-to-up transitions only if spikes are very closely spaced, and only when the frequency of the pair is large enough (3 ≲ Δ *t* ≲ 8 ms for *f* = 1 Hz). In the case of presynaptic spike pairs occurring at 1 Hz, the model predicts depression or up-to-down transitions for all values of Δ *t*. The Δ *t* ranges for which transitions occur become smaller if the presentation frequency of the spike pairs is reduced (presynaptic spike pairs at 0.5 Hz: down-to-up transitions for Δ *t* < 300 ms; postsynaptic spike pairs at 0.5 Hz: up-to-down transitions for 3 ≲ Δ *t* ≲ 6 ms). Nevian and Sakmann found that three postsynaptic spikes at 50 Hz, repeated 60 times at 0.1 Hz, do not evoke any synaptic changes [55]. We find a similar outcome in our model, but predict that an increase in frequency and/or decrease in the burst inter-spike interval should lead to potentiation. This prediction is, however, again sensitive to the extent of summation in calcium in between spikes. If the calcium transients evoked by the back-propagating action potentials do not accumulate, but the second BPAP evokes a calcium transient with the same amplitude as the first one, no down-to-up transitions are observed in the model. We have also investigated the transition behavior of the CaMKII system in response to spike triplets [43], and our model reproduces qualitatively such data provided short-term depression (STD) is added, as in [92] (unpublished data).

In conclusion, our model possesses two stable states of CaMKII activation, which could represent the core mechanism of binary synaptic strength maintenance. We furthermore show that it is possible to reproduce qualitatively experimental STDP results on LTP- and LTD-like transitions. These two results taken together suggest that the CaMKII-associated protein network could account for storage *and* induction of synaptic changes. Our model therefore predicts that the CaMKII protein also plays a major role in LTD, namely that CaMKII gets dephosphorylated during LTD induction. Experiments addressing the role of CaMKII in LTD provide controversial results. Sajikumar et al. showed that LTD in hippocampal CA1 neurons is blocked by CaMKII inhibition during induction but the application of the CaMKII inhibitor (KN-62) after the induction had no impact on LTD [93]. In other experiments, LTD has been shown to occur in the presence of CaMKII inhibitors during LTD induction in hippocampal cultures and slices [21,43]. Such inhibitors bind to CaMKII and block its activation by calmodulin (inhibitor KN-62, which is known not to inhibit the autophosphorylated kinase; used in [43]) or interact with the ATP-binding site of CaMKII (K252a used in [21]) [24]. In the presence of each of both inhibitors, CaMKII can still get dephosphorylated by PP1. We predict that LTD will no longer occur if the CaMKII–phosphatase interaction is disrupted. However, LTD experiments on the hippocampus, the somatosensory cortex, as well as the perirhinal cortex of rats suggest that the metabotropic glutamate receptor (mGluR) pathway is also involved in LTD [55,75,90,94]. The biochemical cascades emerging from mGluR activation could in principle make the occurrence of LTD transitions more robust. The negative coupling of group II mGluRs with the cAMP–PKA pathway [95–97] is consistent with the idea presented here, that LTD requires a shift in kinase–phosphatase balance in favor of phosphatases. Overall, we suggest the dynamics of the global calcium time course play a crucial role for the sign of synaptic changes alongside the crosstalk between signaling cascades that include the one considered here.

## Materials and Methods

### Model of the CaMKII system with constant PP1 activity.

*Calcium binding to calmodulin.* Calmodulin contains four calcium binding sites, two at the C- and two at the N-terminal domain. Calcium binding happens in a cooperative manner in each one of these pairs [98]. The following scheme describes the macroscopic binding of calcium to calmodulin, i.e., we take into account the number of bound calcium ions only, regardless of the occupied microscopic binding sites:

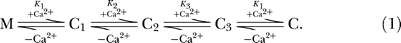



Here M is free intracellular calmodulin and C_i_ (*i* = 1,2,3,4) with C_4_ ≡ C denotes the calcium/calmodulin complex with *i* bound calcium ions. Calmodulin target proteins including CaMKII are partially activated by calmodulin with two, three, or four calcium ions bound. However, CaMKII autophosphorylation rates induced by calmodulin bound with two or three calcium ions are much smaller than with calmodulin bound with four calcium ions [99]. Hence, we consider for simplicity in the model that only calmodulin bound with four calcium ions is able to phosphorylate CaMKII. Since the binding of calcium by calmodulin is fast (with binding rates of the order ∼1000 (*μ*M/s)^−1^ [100,101]), we assume reaction Equation 1 to be in equilibrium with the calcium concentration. The macroscopic dissociation constants of successive calcium binding are taken from Linse et al. (see Table 1A for parameters [100]). The total concentration of calmodulin is *CaM*_0_ = *M* + *C*_1_ + *C*_2_ + *C*_3_ + *C*. Experimental studies suggest that the total available level of calmodulin in neurons is *CaM*_0_ ≈ 10 μM [98,102,103]. Here, we use a smaller value due to the vast number of target proteins of calmodulin besides CaMKII, and the sequestration of calmodulin by neurogranin in spines under resting conditions (see Table 1 and [102,104]). For simplicity, we do not consider the dynamics of calmodulin sequestration by neurogranin, which has been suggested to provide calmodulin during LTP protocols [105]. Assuming a calmodulin bath is an effective way to implement dissociation of calmodulin–neurogranin complexes, which provides calmodulin to the PSD during autophosphorylation and phosphatase/kinase activation. Italic style symbols in this manuscript refer to concentrations of the respective element or protein.

*Autophosphorylation of CaMKII*. The calcium/calmodulin-dependent protein kinase II (CaMKII) holoenzyme has 12 domains, grouped into two clusters each with six functionally coupled subunits [48,49]. CaMKII is activated by Ca^2+^/calmodulin binding to its subunits. Ca^2+^/calmodulin binding to adjacent subunits in the subunit ring stimulates intersubunit autophosphorylation at the residue theronine-286 in the autoregulatory domain (Thr^286^). Autophosphorylation increases CaMKII affinity for Ca^2+^/calmodulin and prolongs activation beyond dissociation of Ca^2+^/calmodulin. In turn, as long as CaMKII stays activated it can bind to the NMDA-R and phosphorylate exogenous substrates [24,49]. For simplicity, some aspects of CaMKII function are not accounted for in the model. Any differences between the CaMKII*α* and *−β* isoforms are not considered. The binding of calcium/calmodulin and protein phosphatase 1 to a subunit is assumed to be independent of the state of neighboring subunits. The autophosphorylation at Thr^305^ and Thr^306^ is not considered.

CaMKII autophosphorylation is an intersubunit process during which one subunit acts as substrate and the neighboring subunit as catalyst. For autophosphorylation to take place, calmodulin has to be bound to the substrate subunit [49]. Autophosphorylation at Thr^286^ or binding of calmodulin each disable the autoinhibitory domain, therefore the catalytic subunit can be in one of the following states: (i) bound with calmodulin, (ii) phosphorylated and bound with calmodulin, or (iii) phosphorylated only (for an illustration see Figure 1C–1E) [27].

The chemical reaction schemes in Figure 1A–1E show schematically how binding of calmodulin and autophosphorylation is represented in the model. Reactions in 1A and 1B show calcium/calmodulin complex binding to dephosphorylated- or phosphorylated subunits, respectively. Autophosphorylation steps where the catalytic subunit is bound with calcium/calmodulin, phosphorylated and bound with calcium/calmodulin, or phosphorylated only are illustrated in Figure 1C, 1D, and 1E, respectively. The intersubunit autophosphorylation is likely to be a directed interaction in the ring and is here assumed to proceed in a single direction [27].

For the autophosphorylation steps depicted in Figure 1C, 1D, and 1E to occur, the substrate subunit must bind the calcium/calmodulin complex C. Let *γ* be the probability that a dephosphorylated subunit S binds with C, i.e., *γ = SC /* (*S + SC*) (SC stands for a dephosphorylated subunit bound with C); and *γ* ^*^ the probability that a subunit phosphorylated at Thr^286^, S^*^, binds with C, i.e., *γ* ^*^ = *S* ^*^ *C* / (*S* ^*^ + *S* ^*^ *C*) (S^*^C stands for a phosphorylated subunit bound with C). Assuming reactions in Figure 1A and 1B to be in equilibrium and using the Law of Mass Action yields *SC* = *S* · *C* / *K*_5_ and S^*^C = S^*^· *C* / *K*_9_, respectively, where *K*_5_ = *k*_−5_ / *k*_5_ and *K*_9_ = *k*_−9_ / *k*_9_ are the dissociation constants of reactions shown in Figure 1A and 1B, respectively. These assumptions lead to:

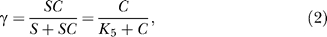


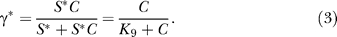



The probability that reaction in Figure 1C takes place in a unit time between two subunits in the single direction is *k*_6_ *γ* ^2^. Correspondingly, the probability for reaction in Figure 1D to occur in a unit time is *k*_7_ γγ^*^ and for reaction in Figure 1E to occur is *k*_8_ *γ* (1 – γ^*^). This probabilistic description of autophosphorylation allows us to describe a given subunit by two possible states only, i.e., whether or not a subunit is phosphorylated at Thr^286^. Note that with a six-subunit ring this yields 14 macroscopic distinguishable activation states (see below). This ansatz does not account for calmodulin consumption during the process of autophosphorylation, assuming a bath of calmodulin. Similar approaches have been used in the investigations of Okamoto and Ichikawa as well as Zhabotinsky in CaMKII models exhibiting bistability [17,18], and in other studies describing in detail CaMKII autophosphorylation, but do not exhibit bistability [106–109].

*Dephosphorylation of CaMKII*. PP1 is the only protein phosphatase that dephosphorylates CaMKII associated with the postsynaptic densities [73]. The dephosphorylation of a free, phosphorylated subunit, and a phosphorylated subunit bound with the calcium/calmodulin complex are described according to the Michaelis-Menten scheme:

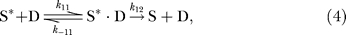


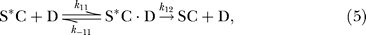
where *D* denotes the concentration of active PP1. Note that dephosphorylation happens independently whether a subunit is bound with the calcium/calmodulin complex or not. Assuming the 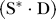 and 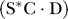 formations are at equilibrium, i.e., 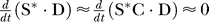 , we can use the standard Michaelis-Menten equation [110] to obtain the per-subunit rate of dephosphorylation, *k*_10_,

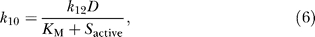
where the Michaelis constant *K*_M_ is given by *K*_M_ = (*k_−_*_11_ *+ k*_12_) / *k*_11_ and *S*_active_ is the total concentration of phosphorylated CaMKII subunits, 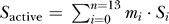 , where *m_i_* is the number of phosphorylated subunits of the macroscopic activation state *i* (see below for more details). The per-subunit rate of dephosphorylation, *k*_10_, is proportional to the amount of available phosphatase, *D*. The dephosphorylation rate *per* subunit declines if a lot of subunits are phosphorylated and the phosphatase activity remains constant, i.e., if *S*_active_ is high and *D* constant. This saturation of *k*_10_ leads to the bistable behavior of the CaMKII phosphorylation level (see “Bistability of the CaMKII system with constant PP1 activity” section).

Applying the Law of Mass Action and taking into account the geometry of the CaMKII six-subunit ring, its autophosphorylation and dephosphorylation by PP1 is described by the following system of coupled, ordinary differential equations for concentrations of CaMKII with different numbers of phosphorylated subunits

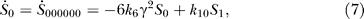


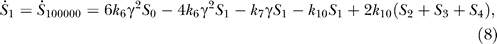


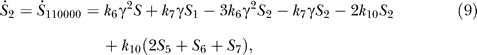


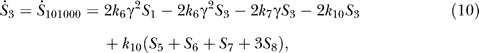


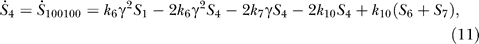


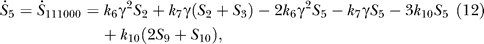


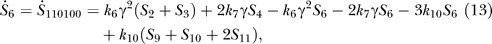


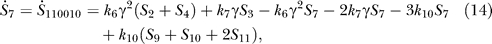


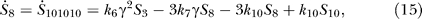


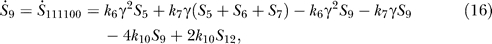


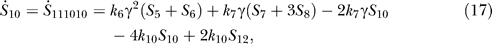


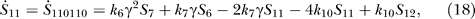


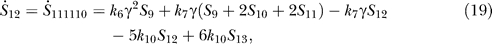


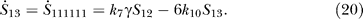



Here *S_i_* refers to the concentration of the 14 (*i* = 0,...,13) macroscopic distinguishable activation states of the CaMKII protein. The subscript in the second column shows the geometrical order of Thr^286^ phosphorylated sites in the CaMKII ring, 1 refers to a phosphorylated subunit, 0 to a dephosphorylated subunit. Attention should be drawn to the fact that, for example, *S*_5_, *S*_6_, *S*_7_, and *S*_8_, all have three phosphorylated subunits, i.e., all of them have the same macroscopic level of activation, i.e., *m*_5_ = *m*_6_ = *m*_7_ = *m*_8_ = 3. However, in terms of symmetry all four have to be distinguished since at *S*_5_ the phosphorylated sites are adjoined, *S*_111000_, whereas at *S*_8_ they are separated by a dephosphorylated subunit, *S*_101010_, for example. With regard to this difference, the propagation of autophosphorylation is different for both, the phosphorylation step shown in Figure 1C can occur on two pairs of subunits at *S*_5_ but cannot occur at *S*_8_ at all. Taking into account that the different autophosphorylation steps, depicted in Figure 1C–1E, happen with different probabilities leads to differing occurrences of the activation states *S_i_* (with *i* = 0...13). Note that we used the fact *k*_7_ = *k*_8_ and simplified *k*_7_ γγ^*^ + *k*_8_ *γ* (1 – γ^*^) to *k*_7_ *γ*. *k*_10_ is the per-subunit rate of dephosphorylation (see above).


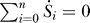
 , with *n* = 13 for 14 macroscopic distinguishable activation states of the six-subunit CaMKII ring, yields the CaMKII protein mass conservation, 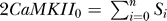 . *2CaMKII*_0_ gives the total concentration of functionally independent CaMKII clusters of six subunits and *CaMKII*_0_ the total CaMKII concentration since one holoenzyme comprises two six-subunit rings. Note that the number of macroscopic distinguishable activation states is 3, 6, 14, and 36 for the two, four, six, and eight subunit models, respectively.

### Model with Ca-dependent PP1 activity via protein signaling cascade including PKA and calcineurin

The dephosphorylation activity of PP1 is indirectly governed by calcium/calmodulin via inhibitor 1 (I1), i.e., phosphorylated inhibitor 1 inhibits PP1 [111,112]. Inhibitor 1 itself is phosphorylated by cyclic AMP-dependent protein kinase A (PKA) and protein kinase G and dephosphorylated by the phosphatase calcineurin and protein phosphatase 2A [37,38,60,70–72]. A simple realization of this protein signaling cascade is given by

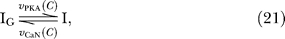


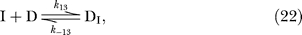
where I_G_ refers to dephosphorylated I1, I denotes phosphorylated inhibitor 1 (I1P), D is free PP1, and D_I_ stands for inhibited PP1, i.e., PP1 bound with phosphorylated inhibitor 1. See Figure 1F for a scheme of the protein signaling cascade.

The balance between inhibitor 1 phosphorylation (*v*_PKA_)- and dephosphorylation rate (*v*_CaN_) is calcium/calmodulin-dependent. The enzymatic activity of calcineurin can be described by a Hill equation [113]. The PKA activity is also known to be calcium/calmodulin-dependent via cyclic AMP [69], but there is no data characterizing this dependency. We chose to describe both by a Hill equation

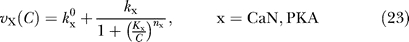
with a calcium/calmodulin-independent base activity (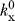 ) which also accounts for protein kinase G phosphorylation (x = PKA) and protein phosphatase 2A dephosphorylation (x = CaN). *k*_x_ is the maximal, calcium/calmodulin-dependent activity, *K*_x_ the half activity concentration, and *n*_x_ denotes the Hill coefficient.

Applying the Law of Mass Action and taking into account protein phosphatase 1 conservation yields

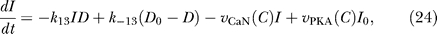


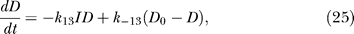
where *I*_0_ and *D*_0_ = *D* + *D*_I_ refer to the total I1 and PP1 concentration, respectively. The concentration of dephosphorylated, free inhibitor 1, *I*_0_, is treated like a bath, assuming a rapid exchange between the PSD with the spine volume and between the spine and the parent dendrite, as in [17,57]. Therefore, inhibitor 1 is not conserved in Equation 24 due to this bath assumption.

### Approximation of the PP1 activity level after presentation of one spike pair.

As can be seen in Figure 5C and 5D, the change in PP1 activity, as well as the change in I1P concentration (unpublished data), during the presentation of one spike pair is small. We therefore separate both variables into two terms, a constant value and a small time-dependent change, i.e., *D*(*t*) → D^*^ + *ɛδD*(*t*) and *I*(*t*) → I^*^ + *ɛδI*(*t*), where D^*^ and I^*^ are the values before the spike-pair presentation, and *δD*(*t*) and *δI*(*t*) describe the changes during the presentation. Since these small changes are exclusively driven by changes in *v*_CaN_(*t*) and *v*_PKA_(*t*), we consider the time-dependent part of both rates as small compared to *k*_13_ and *k*_−13_, i.e., 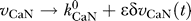 and 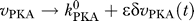 . Inserting these expressions in Equations 24 and 25 yields at zero order in *ɛ* the steady-state values D^*^ and I^*^. The first-order equations in *ɛ* are

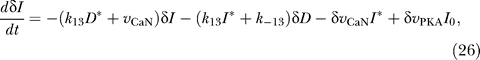


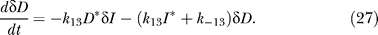



The Eigenvalues of the homogeneous system of Equations 26 and 27 are

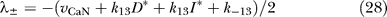


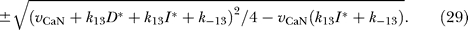



Since *v*_CaN_ is much smaller than *k*_13_ *D* ^*^, *k*_13_ *I* ^*^, or *k*_−13_, we expand the two Eigenvalues around *v*_CaN_. This yields a fast and a slow Eigenvalue since *λ_+_* is zero at leading order. The Eigenvalues become

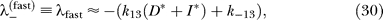


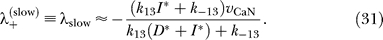



With the initial conditions *δD*(0) = *δI*(0) = 0, the solution for the inhomogeneous system (Equations 26 and 27) becomes

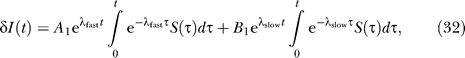


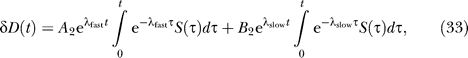
with *A*_1_ = −(*k*_13_ *D* ^*^ + *v*_CaN_ + *λ*_slow_) / (*λ*_fast_ − *λ*_slow_), *B*_1_ = (*k*_13_ *D* ^*^ + *v*_CaN_ + *λ*_fast_) / (*λ*_fast_ − *λ*_slow_), *A*_2_ = −*k*_13_ *D* ^*^ / (λ_fast_ − λ_slow_), and *B*_2_ = *k*_13_ *D* ^*^ / (*λ*_fast_ − *λ*_slow_). *S*(τ) is the inhomogeneous part in Equation 26, i.e., *S*(*τ*) = −*δv*_CaN_ (*τ*)*I* ^*^ + *δv*_PKA_(*τ*)*I*_0_. The first term in Equations 32 and 33 describes the fast dynamics of both variables and allows *D* and *I* to follow on a fast time scale the calcium transient. After the spike-pair presentation, this term decays rapidly with the time constant *λ*_fast_. The second term determines the slow dynamics of the system and therefore gives rise to a slow buildup, which decays after the spike-pair presentation with the slow time constant *λ*_slow_. Since (*λ*_slow_ · *t*) is small at the scale of single presentations, we obtain for the slow dynamics

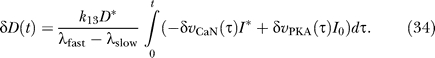



*δD*(*t*) is shown in Figure 9B as a measure for the slowly decaying PP1 buildup after the presentation of one spike pair. *D* ^*^ + *δD*(*t*) is compared with the PP1 activity obtained from numerical integration of Equations 24 and 25 after one spike pair in Figure 9A. Note that the product of *δD*, *D* ^*^, and *D* with *k*_12_ is shown in Figure 9A and 9B.

In the section “STDP protocol with deterministic calcium transients,” we point out that an increase in *R* beyond the value of 1 does not significantly affect the dynamics of the PP1 response, which is basically determined by 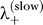 (see paragraph above). This can be understood by considering 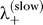 (Equation 31), if *D* ^*^≪ *I* ^*^, *k*_−13_ / *k*_13_, its denominator, will be controlled by *k*_13_ *I* ^*^ and *k*_−13_ only, and changes in *D* ^*^ will have no impact on the PP1 dynamics.

### Synaptic activity and postsynaptic calcium signaling.

To investigate how the model behaves when realistic calcium transients are applied to it, we use the following model for postsynaptic calcium and postsynaptic membrane potential dynamics. We focus on a single spine compartment, and do not simulate the backpropagation of the action potential from its initiation site to the spine. Instead, we model the action potential dynamics directly at the spine.

*Postsynaptic membrane potential*. The postsynaptic membrane potential is modeled using the Hodgkin-Huxley formalism in a single compartment. The reference volume for the membrane potential and the calcium dynamics model is taken to be a postsynaptic spine (*V*_spine_ ≈ 1 μm^3^, *r*_spine_ ≈ 0.5 μm). The dynamics of the membrane potential *V* follows the differential equation

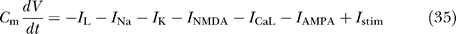
where *C*_m_ is the whole cell capacitance of 0.1 nF, *I*_x_ (x = L, Na, K, NMDA, CaL, AMPA) are the ionic currents listed below. An action potential is evoked by a 1 ms depolarizing pulse current *I*_stim_ of 3 nA.

*Postsynaptic calcium dynamics*. The model of the calcium dynamics involves the two main sources of postsynaptic calcium influx in the spine: NMDA receptors (NMDA-R) and voltage-dependent calcium channels (VDCC) [114]. Extrusion, diffusion, and slow buffering is accounted for by a single exponential decay, yielding the following equation for the time course of the intracellular calcium concentration

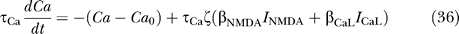
where *Ca* is the free, intracellular calcium concentration, *τ*_Ca_ = 12 ms refers to the single exponential time constant of the passive decay process [54], *Ca*_0_ is the calcium resting concentration, and *ζ* = 2.59 · 10^4^ m^2^ μM/C converts the ion currents into concentration changes per time for a spine of volume ≈1 μm^3^. *I*_x_ (x = NMDA, CaL) are the ionic currents listed below. Scaling parameters *β*_NMDA_ = 1/1000 and *β*_CaL_ = 1/100 take into account both the immediate uptake of calcium by intracellular buffers (∼99%, [115]) and the fact that only about ∼10% of the NMDA-mediated current is carried by calcium ions (see [116,117] and below).

*Noisy calcium transients*. To investigate stochastic effects, we add two possible noise sources to the model: (i) NMDA receptor maximum conductance drawn at random at each presynaptic spike and (ii) maximum conductance of the voltage-dependent calcium channel drawn at random at each postsynaptic spike. Both conductances are drawn from binomial distributions characterized by the total number of channels *N*_tot_ and the opening probability per channel *p*_o_. Each presynaptic or postsynaptic spike gives rise to an integer number, *n*_o_, of NMDA or CaL channel openings, respectively. We assume that the channels open independently of each other. The single channel conductance *g*_single_ is chosen so that the mean calcium amplitudes are as stated above. To account for the stochasticity of calcium ions influx, Gaussian noise with zero mean and a variance scaled with *n*_o_ is added to (*n*_o_ · *g*_single_). The parameters of the NMDA and CaL maximum conductance distributions are adjusted such that they fit the experimental findings of single spine measurements by Mainen et al. and Sabatini and Svoboda, respectively [58,59] (see Table 2 for parameters).

*Ion currents dynamics*. The description and the parameters of the ionic currents are taken from Poirazi et al. (*I*_CaL_) as well as Purvis and Butera (*I*_Na_, *I*_K_) [118,119].

Leak Current: The leak current is given by

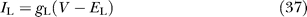
where *g*_L_ is the leak conductance. The leak potential is adjusted such that the resting potential is −70 mV.

The ionic currents listed here have the general form *I*_ionic_ = *gy*(*V – E*_ionic_). *E*_ionic_ is the reversal potential for the respective ions carried, *g* refers to the maximum conductance of each current, and *y* is the product of one or more gating variables. *y* determines the dynamics of the ion currents regulated by voltage-dependent activation and inactivation variables which are described according to

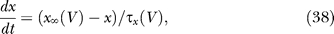


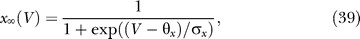


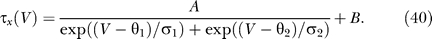



Here *x*_∞_(*V*) is the steady-state voltage-dependent (in)activation function of *x*, and *τ_x_*(*V*) is the voltage-dependent time constant. In terms of this formalism, the respective ion currents are given by:

Sodium current:

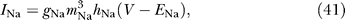


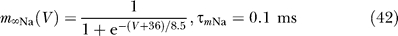


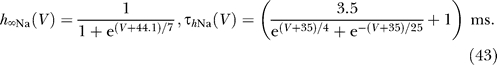

Delayed-rectifier Potassium current:

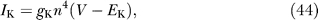


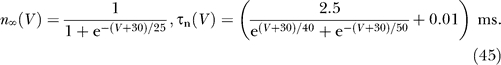

Voltage-dependent calcium current (high-voltage activated L-type):

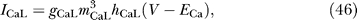


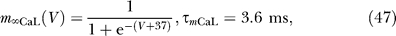


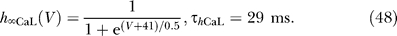



AMPA current: excitatory postsynaptic potentials are mainly mediated by the AMPA receptor current given by

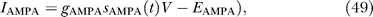


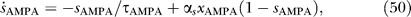


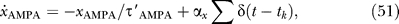
with *τ*_AMPA_ = 2 ms, 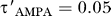 ms, *α_s_* = 1 1/ms, and *α_x_* = 1 (dimensionless) [120,121]. *s*_AMPA_ is a single exponentially decaying gating variable with a finite rise time (the time-to-peak is ≈0.2 ms). At each occurrence of a presynaptic spike at time *t_k_*, the variable *x*_AMPA_ is increased by one (the sum on the right-hand side of Equation 51 goes over all presynaptic spikes occurring at times *t_k_*).

NMDA current: the current mediated by the NMDA receptor is described by

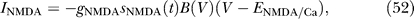
where the voltage dependence of the magnesium block is given by

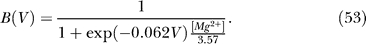



The voltage dependence is controlled by the extracellular magnesium concentration [*Mg* ^2+^] = 1.0 mM [122]. The dimensionless gating variable *s*_NMDA_ obeys the same types of equations as *s* and *x* of the AMPA current (Equations 50 and Equation 51, respectively) but with *τ*_NMDA_ = 80 ms and 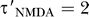 ms [121] (the time-to-peak is ≈8 ms).

The maximum leak conductance and the whole cell capacitance yield a membrane potential time constant *τ*_m_ of 20 ms, according to the equation *τ*_m_ = *C*_m_ / *g*_L_. The AMPA receptor conductance *g*_AMPA_ is chosen such that a single presynaptic spike evokes a maximal depolarization of 1 mV at −70 mV. *g*_NMDA_ and *g*_CaL_ are chosen such that the amplitudes of the NMDA-R mediated and the action potential–evoked calcium transients in the spine are realized as stated in the text. The ratio of ∼2 between the BPAP evoked calcium transient amplitude (Δ*Ca*_post_) and the NMDA-R mediated contribution (Δ*Ca*_pre_) is as measured by Sabatini et al. [54]. Note that the VDCC- and the NMDA-mediated calcium currents in the calcium dynamics (Equation 36) are multiplied by the scaling parameters *β*_NMDA_ and *β*_CaL_, which account for fast calcium buffering and for the fractional calcium current through NMDA-Rs of ∼10 %. The calcium reversal potential, *E*_Ca_, is used to describe the fractional calcium current through NMDAs in the calcium dynamics (Equation 36), whereas the reversal potential of the compound sodium, potassium, and calcium ion current, *E*_NMDA_, mediated by NMDA-Rs, is employed in the voltage equation (Equation 35).

*Parameters of the model*. The model describing the interactions between proteins contains a large number of parameters (25). In some cases, we used experimentally determined values (see Table 1A for a list of those parameters). Other parameters are not (or poorly) determined experimentally. These parameters were varied systematically or were determined by the constraints we impose on the model (see Table 1B). Finally, a few parameters were set on the basis of previous modeling studies or set to an arbitrary value, in cases in which changing this value does not alter the results of the model (see Table 1C).

The calcium-dependent steady-state concentration of phosphorylated CaMKII subunits depends heavily on the choice of the parameters defining the PKA pathway (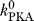 ,*k*_PKA_, *K*_PKA_, *n*_PKA_). These parameters are adjusted in order to obtain the “LTD” and the “LTP window” at specific intervals of calcium concentration (see section “LTD window” in a model with Ca-dependent PP1 activity via protein signaling cascade including PKA and calcineurin). The maximal calcineurin activity *k*_CaN_ is used to adjust the PP1 level evoked during the STDP stimulation protocol (see section “STDP protocol stimulation with stochastic calcium dynamics”).

The total calmodulin concentration (*CaM*_0_) is smaller than the value found in experimental studies, due to the reasons given above (see “Calcium binding to calmodulin” paragraph). *K*_M_ is taken from the modeling study of [17]. Equation 24 and 25 give the steady-state PP1 concentration, *D*_steady-state_ = *D*_0_ / (1 + (*I*_0_ *k*_13_ *v*_PKA_) / (*k_−_*_13_ *v*_CaN_)). Hence, *D*_steady-state_ depends on *I*_0_, *v*_PKA_, and *v*_CaN_ through the single variable 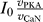 . This means that out of the five parameters *I*_0_, 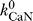 , *k*_CaN_, 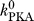 , and *k*_PKA_, the steady-state PP1 concentration depends on three independent combinations of those parameters, e.g., 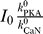 , 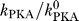 , and 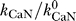 . Thereby, two out of these five parameters can be set arbitrarily. The total I1 concentration and the calcineurin base activity, *I*_0_ and 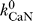 , are set to the values given in Table 1C and are kept constant throughout all investigations, while the remaining three parameters *k*_CaN_, 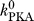 , and *k*_PKA_ are obtained by constraints imposed on the model (see Table 1B). On the other hand, the dynamics of the protein signaling cascade depends on all five parameters. We address this issue via the scaling parameter *R* which influences the PP1 response dynamics (see “STDP protocol stimulation with stochastic calcium dynamics” section).

The parameters describing postsynaptic calcium and postsynaptic membrane potential dynamics are taken from previous modeling studies [59]. We systematically vary the calcium amplitudes evoked by a presynaptic (Δ*Ca*_pre_) and a postsynaptic spike (Δ*Ca*_post_), keeping their ratio constant, Δ*Ca*_post_ = 2 · Δ*Ca*_pre_ (see the section “Effect of Kinetics of Autophosphorylation and Dephosphorylation on the Number of Spike-Pair Presentations Needed for Transitions”).

### Numerical methods.

We solve the system of coupled, ordinary differential equations with a fourth-order Runge-Kutta method with adaptive stepsize control. This has been implemented in a C++ program. We used XPPAUT by G. Bard Ermentrout (http://www.pitt.edu/~phase/) for the steady-state calculations of the CaMKII system.

## Abbreviations

AMPA: α-amino-3-hydroxyl-5-methyl-4-isoxazole-propionate acid
CaMKII: calcium/calmodulin-dependent protein kinase II
cAMP: cyclic adenosine monophosphate
LTD: long-term depression
LTP: long-term potentiation
PKA: protein kinase A
PP1: protein phosphatase 1
PSD: postsynaptic density
STDP: spike-timing dependent plasticity
